# Comparative metabolomics of *Phialemonium curvatum* as an omnipotent fungus cultivated on crude palm oil *versus* glucose

**DOI:** 10.1186/s12934-020-01434-w

**Published:** 2020-09-09

**Authors:** Arief Izzairy Zamani, Susann Barig, Sarah Ibrahim, Hirzun Mohd. Yusof, Julia Ibrahim, Jaime Yoke Sum Low, Shwu Fun Kua, Syarul Nataqain Baharum, Klaus-Peter Stahmann, Chyan Leong Ng

**Affiliations:** 1 Institute of Systems Biology, Universiti Kebangsaan Malaysia (UKM), 43600 Bangi, Selangor Malaysia; 2 Institute of Biotechnology, Brandenburg University of Technology Cottbus –Senftenberg, Universitaetsplatz 1, 01968 Senftenberg, Germany; 3 Sime Darby Technology Centre, 1st Floor Block B, UPM-MTDC Technology Centre III, Lebuh Silikon, UPM 43400 Serdang, Selangor Malaysia

**Keywords:** *Phialemonium curvatum*, Omnipotent fungus, Comparative metabolomics, Central carbon metabolism, Selective minimal media

## Abstract

**Background:**

Sugars and triglycerides are common carbon sources for microorganisms. Nonetheless, a systematic comparative interpretation of metabolic changes upon vegetable oil or glucose as sole carbon source is still lacking. Selected fungi that can grow in acidic mineral salt media (MSM) with vegetable oil had been identified recently. Hence, this study aimed to investigate the overall metabolite changes of an omnipotent fungus and to reveal changes at central carbon metabolism corresponding to both carbon sources.

**Results:**

Targeted and non-targeted metabolomics for both polar and semi-polar metabolites of *Phialemonium curvatum* AWO2 (DSM 23903) cultivated in MSM with palm oil (MSM-P) or glucose (MSM-G) as carbon sources were obtained. Targeted metabolomics on central carbon metabolism of tricarboxylic acid (TCA) cycle and glyoxylate cycle were analysed using LC–MS/MS-TripleQ and GC–MS, while untargeted metabolite profiling was performed using LC–MS/MS-QTOF followed by multivariate analysis. Targeted metabolomics analysis showed that glyoxylate pathway and TCA cycle were recruited at central carbon metabolism for triglyceride and glucose catabolism, respectively. Significant differences in organic acids concentration of about 4- to 8-fold were observed for citric acid, succinic acid, malic acid, and oxaloacetic acid. Correlation of organic acids concentration and key enzymes involved in the central carbon metabolism was further determined by enzymatic assays. On the other hand, the untargeted profiling revealed seven metabolites undergoing significant changes between MSM-P and MSM-G cultures.

**Conclusions:**

Overall, this study has provided insights on the understanding on the effect of triglycerides and sugar as carbon source in fungi global metabolic pathway, which might become important for future optimization of carbon flux engineering in fungi to improve organic acids production when vegetable oil is applied as the sole carbon source.

## Background

Glucose is a common carbon source in fermentation. Recently, several studies have been done on plant triglycerides or vegetable oil to replace glucose in fermentation [1–5]. For instance, palm oil was found to not only replace lactose but led to a better cell growth and penicillin production in *Penicillium chrysogenum* culture [3]. Soybean oil was explored and successfully used to culture *Ashbya gossypii* for the better production of riboflavin [1]. Recently, *Phialemonium curvatum* AW02 was shown to grow well in acidic (pH 2 – 3) minimal media with rapeseed oil as the carbon and energy source [6]. These studies showed that plant triglycerides could serve as an alternative carbon source for microbial growth. Knowing that only certain lipase-secreting microorganisms are able to grow on this carbon source, triglycerides is selected as the carbon source in selective minimal media. It can suppress the growth of unwanted bacterial contaminant since they are unable to hydrolyse the plant triglyceride [6].

*P. curvatum* AW02 has been shown to generate an extracellular lipase, even active at acidic pH of 3 [6]. Lipases which are active at acidic pH are found in mammals but are rare in microorganisms [7]. Recently, *P. curvatum* AW02 was found to produce several secondary metabolites including 4-hydroxybenzoic acid, a commercial metabolite used as drugs preservative [8]. Furthermore, the study also identified 3-indole acetic acid which is a well-known plant hormone and solaniol, a naphthoquinone derivative [8]. These growing number of studies on *P. curvatum* highlighted its significance in biotechnology.

Vegetable oil including palm oil is known to contain triglyceride, free fatty acid, and vitamin. Triglycerides are the major component in vegetable oils which consist about 95 – 98% [9]. Genera of filamentous fungi comprise of *Aspergillus*, *Rhizopus*, *Penicillium*, *Mucor*, *Geotrichum*, and *Fusarium* were shown able to degrade triglycerides into free fatty acids through hydrolysis process with extracellular lipases [10]. The liberated free fatty acids can be taken by the microorganism and undergo β-oxidation to produce acetyl-CoA [1]. The acetyl-CoA will then take part in glyoxylate cycle which functions to synthesize malate and via PEP to produce all anabolites which will be needed [11] when glucose is depleted [12]. On the other hand, fatty acid is highly reduced and therefore known to provide six times more energy when compared to polysaccharide if the bound water is taken into consideration [13]. In the central carbon metabolism understanding, many studies have provide insights on the organic acids concentration in TCA cycle for several fungal species grown on glucose [14, 15]. On the contrary, the details on concentration for each organic acid in the glyoxylate pathway is still lacking, although extensive studies have showed that glyoxylate pathway is activated in many fungal species that grown on non-sugar carbon sources [16–19].

Hereby, taking the advantage of *P. curvatum* AW02 that able to grow on sugar or plant triglycerides in minimal media, this study aimed to provide the details on the changes in central carbon metabolism for the species. In addition, systematic comparative interpretation of metabolic changes is also possible with current metabolomics platform. Hence, the analysis of metabolic changes for both non-targeted metabolites and targeted organic acids which involved in tricarboxylic acid (TCA) cycle or glyoxylate pathway of *P. curvatum* AW02 cultivated in minimal media with palm oil replacing glucose as sole carbon source was conducted. For the first time, the effect of triglycerides replacing sugar as carbon source on fungal metabolism was investigated using a systematic comparative metabolomics approach. The findings have provided insights that are important for the development of metabolic engineering, in particular on organic acids production of omnipotent fungi that grow on vegetable oil.

## Results

### Growth comparison of *P. curvatum* AW02 in MSM-P and MSM-G media

**Fig. 1:**
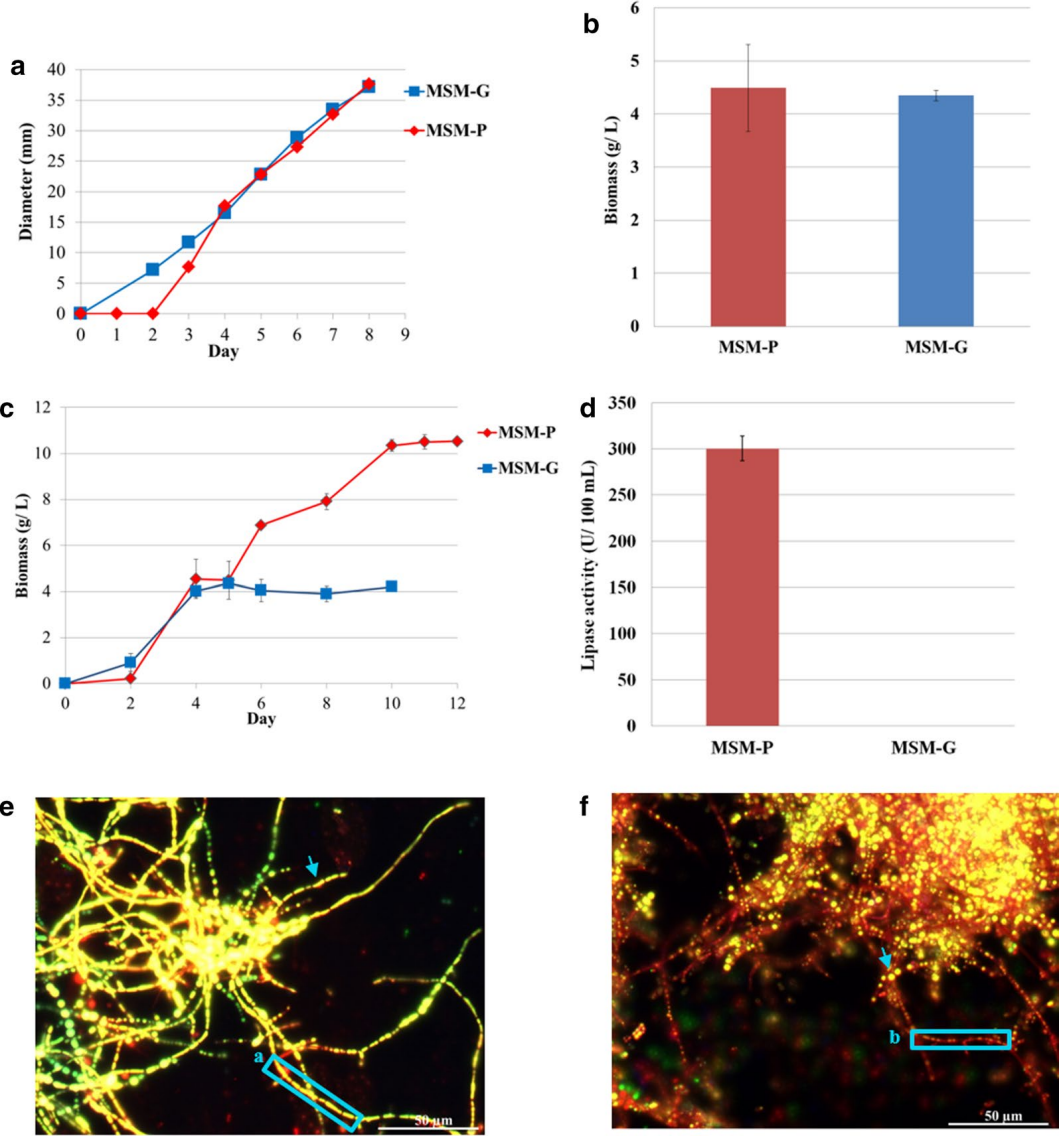
**a** Colony diameter profile of *P. curvatum* AW02 on MSM-P agar and MSM-G agar at 33 °C. Colony diameters were determined every 24 h. Values are given as mean of three independent experiments. Standard error was below 20%. **b** Biomass of *P. curvatum* AW02 after 5 days culture in MSM-P and MSM-G broth. **c** Biomass profile of *P. curvatum* AW02 cultivated in MSM-P and MSM-G. **d** Lipase activity at day 5 from media of MSM-P or MSM-G cultured with *P. curvatum* AW02. Lipid droplets inside the cells of *P. curvatum AW02* after 5 days cultured in media: **e** Cells harvested from MSM-P culture; **f** Cells harvested from MSM-G culture. Cells were stained with Nile red and observed using Axiostar fluorescence microscope equipped with filter combination of excitation filter BP 550/25 and 470/40 nm. Final magnification was ×400. The arrows indicate the lipid bodies. The box (a) showed the abundance of lipid bodies with elongated shape in hyphae from MSM-P culture while box (b) showed the scattered and rounded lipid bodies in hyphae from MSM-G culture

*P. curvatum* AW02 showed similar growth rate on MSM-P and MSM-G agar, except the growth for MSM-G culture can be measured starting at day 2, as compared to MSM-P culture at day 3 (Fig. 1a). Nonetheless, similar growth rate of *P. curvatum* AW02 on MSM-P and MSM-G agar indicates that palm oil is an efficient carbon source comparable to glucose (Table 1).

**Table 1:** Growth rate on complex and minimal agar media of *P. curvatum* AW02

Media	Kr, mm day^−1a^
Rich medium	
Glucose	5.7 ± 1
MSM	
Glucose	5.5 ± 0
CPO	5.5 ± 0

*P. curvatum* AW02 were cultured on agar plates containing different agar media. Radial growth was determined every 24 h over 6 days and growth rates were calculated. Crude palm oil (CPO); Mineral salts medium (MSM)

^a^; Results are the mean of 3 replicates ± standard deviation. The values in standard deviation were rounded to integer

Biomass production of *P. curvatum* AW02 in both MSM-P and MSM-G were found similar (4.49 ± 1.6 g/L and 4.35 ± 0.2 g/L, respectively after 5 days of cultivation (Fig. 1b). Nonetheless, prolonged cultivation of *P. curvatum* AW02 in MSM-P up to stationary phase (12 days) produced 10 g/L of biomass, which is 2.5-fold higher as compared to MSM-G culture, which started its stationary phase at day 6 (Fig. 1c). Palm oil as carbon source in defined media has been used for various microorganism cultivation and was found to produce high biomass. For example, *Penicillium chrysogenum* Hl107 was grown up to 10 g/L at 28 °C on basal media [3].

### Lipase activity of *P. curvatum* AW02 in MSM-P culture

Lipase activity was detected in MSM-P culture. However, when glucose was used as carbon source, no lipase activity was detectable (Fig. 1d). Since the detection limit was < 3 U per 100 mL, an induction factor of the respective lipase gene of at least 100 can be concluded.

### Lipid bodies visualization

*P. curvatum* AW02 culture in MSM-P was found to pose abundance of lipid bodies with elongated shape compared to MSM-G culture (Fig. 1E and F). This observation suggested that free fatty acid from palm oil that had been liberated by the fungal lipase was likely uptaken and accumulated in *P. curvatum* AW02 as triglyceride containing lipid bodies in intracellular compartment. The abundance of lipid bodies in intracellular compartment of MSM-P culture may contribute to the high biomass production. This was supported by total cellular lipid quantification, in which the amount of cellular lipids in MSM-P culture was found 13-fold higher in compared to MSM-G culture (Additional file 1: Fig. S1). It has been known that cells can accumulate the fatty acids and form lipid bodies under high carbon concentration and thus increase the cell biomass [20]. Free fatty acid is known to undergo β-oxidation to produce acetyl-CoA for carbon metabolism in TCA cycle [21]. It is possible that the liberated free fatty acid from palm oil might be accumulated and used to synthesise lipid in intracellular compartment as energy storage.

### Untargeted metabolite profiling

**Fig. 2:**
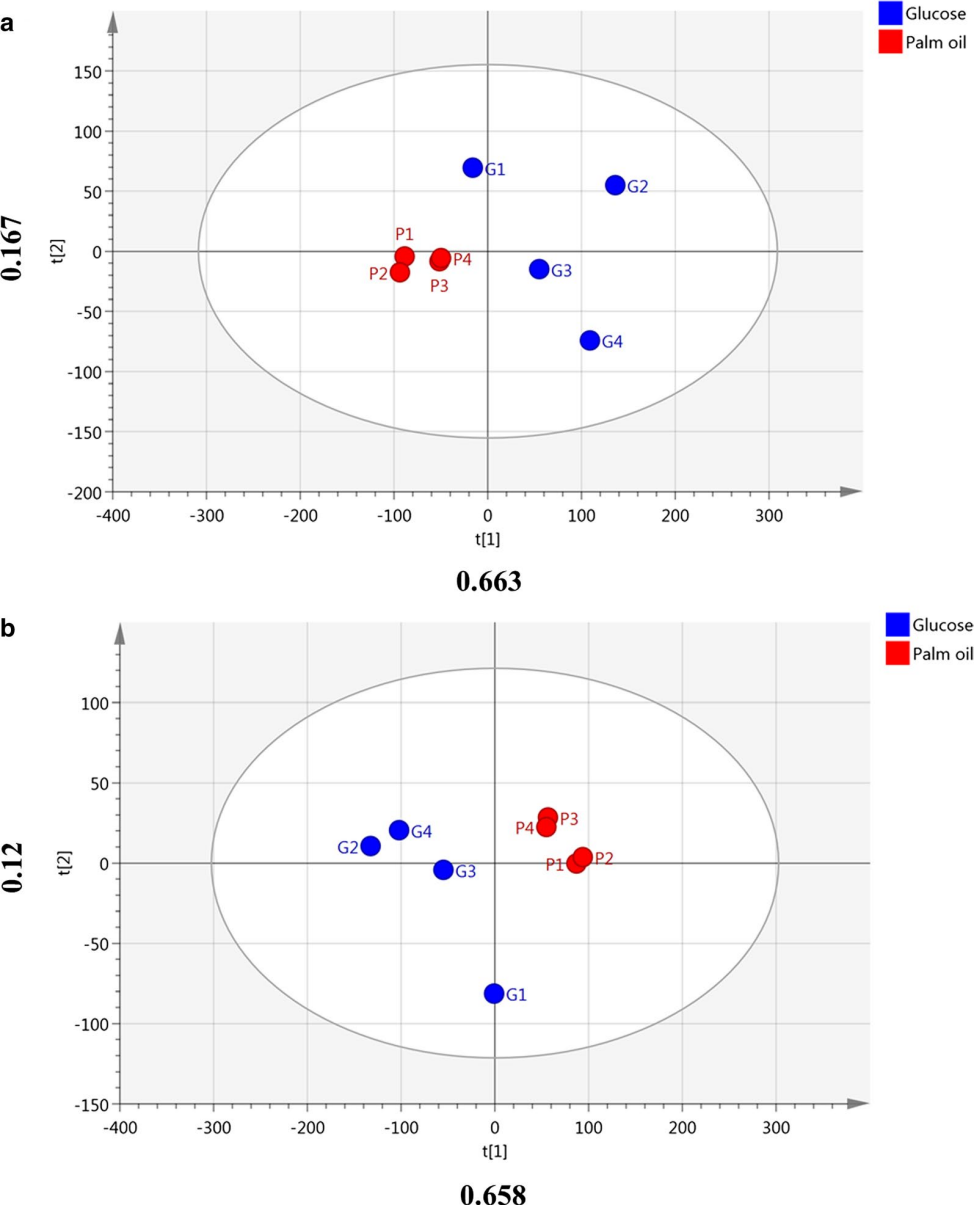
Score plot projection based on intracellular metabolites from MSM-P and MSM-G cultures. **a** PCA (R2X = 0.83, Q2(cum) = 0.469) and **b** PLS-DA (R2X = 0.778, Q2(cum) = 0.738, R2Y = 0.967)

Untargeted profiling of intracellular metabolites of both MSM-P and MSM-G culture using LC–MS-TOF had detected 144 metabolites based on FMF algorithm (Additional file 1: Table S1), with 30 metabolites were successfully identified by cross-checking with QC (Table 2, Additional file 1: Fig. S2). These metabolites that belongs to the classes of sugars, lipids, nucleotides, organic acids, and coenzymes were subjected for multivariate analysis to compare the metabolome differences of MSM-P and MSM-G culture. Principal component analysis (PCA) score plot with a good fit of R2X (0.83) clustered MSM-P and MSM-G into two separate groups (Fig. 2a), indicating that *P. curvatum* AW02 undergoes metabolic changes when palm oil replaces glucose as carbon source. This observation was validated using QC with the intensity’s deviation was < 2SD as shown in Additional file 1: Fig. S3.

**Table 2:** Identification of intracellular metabolites in *P. curvatum* AW02 grown using palm oil and glucose as carbon source

N	RT m/z value	Ion adduct	Major Fragments	Molecular formula	Mass Error (ppm)	Metabolite identification	Id. level^a^	Reference	Class	Trend Palm oil/Glucose	Fold change	^b^VIP	p-value	FDR
1	2.48 min 387.115 m/z	[M−H + CHO2H]–	179.053, 341.101, 161.038	C12H22O11	1	*Trehalose	1	Standard	alpha-linked disaccharide/monosaccharide	Down	− 4.0	3.03	1.22 × 10^−8^	4.65 × 10^−7^
2	2.47 min 729.233 m/z	[M−2H + CHO2H]–	341.102, 387.106, 342.105, 179.052	C12H22O11	5	*Trehalose	1	Standard	alpha-linked disaccharide/monosaccharide	Down	− 16	2.46	7.54 × 10^−5^	0.000915
3	7.97 min 329.234 m/z	[M−H]–	329.227, 285.198, 330.230, 311.220	C18H33O5	2	*Unknown	4	N.A	N.A	Up	21	1.72	5.78 × 10^−5^	0.000915
4	2.45 min 227.077 m/z	[M−H + CHO2H]–	n/a	C6H14O6	1	Mannitol, d-Sorbitol, d-Iditol, l-Iditol, Galactitol, l-Glucitol	3	MetLin	Sugar alcohol	Down	− 3.0	1.41	0.008242	0.019553
5	8.02 min 399.277 m/z	[M−H]–	n/a	C28H36N2	8	*2,5-Bis(4-hexylphenyl)pyrimidine, Pyrimidine, 5-heptyl-2-(4′-pentyl[1,1′-biphenyl]-4-yl)-,	3	MetLin	Pyrimidine derivative	Down	− 6.0	1.05	0.000991	0.003423
6	7.69 min 452.280 m/z	[M−H]–	255.231, 196.036, 385.296	C28H39NO4	1	Unknown	4	N.A	N.A	Down	− 4.0	1.05	0.007892	0.019553
7	2.37 min 302.101 m/z	[M−H + CHO2H]–	168.035, 242.075, 152.989, 169.039	C8H21NO6P	1	*Glycero-phosphocholine	2	MetLin	Lipid	Down	− 3.0	1.01	0.001922	0.006087
8	3.80 min 333.061 m/z	[M−H]–	152.991, 241.006, 171.000	C9H19O11P	5	*sn-Glycero-3-phospho-1-inositol	2	MetFrag	Lipid	Down	− 11	1.00	9.63 × 10^−05^	0.000915
9	4.05 min 191.020 m/z	[M−H]–	111.00, 105.32	C6H8O7	1	*Citric acid	1	Standard	Organic acid	Down	− 9.0	0.97	0.000193	0.00124
10	2.45 min 181.071 m/z	[M−H]–	n/a	C6H14O6	4	Mannitol, d-Sorbitol, d-Iditol, l-Iditol, Galactitol, l-Glucitol	3	MetLin	Sugar alcohol	Down	− 2.0	0.76	0.015403	0.027873
11	8.18 min 599.529 m/z	[M−H]–	n/a	C32H68N6O4	10	Oxalic acid–N″-tetradecylguanidine (1/2)	3	MetLin	Organic acid derivative	Down	− 2.0	0.76	0.25175	0.31888
12	2.49 min 431.106 m/z	[M−H]–	n/a	C24H20N2O4S	3	Benzenamine, 3,3′-[sulfonylbis(4,1-phenyleneoxy)]bis-	3	MetLin	Organic compound	Down	− 5.0	0.74	0.000979	0.003423
14	2.35 min 403.152 m/z	[M−H]–	n/a	C20H24N2O7	2	Myxochelin A, Desmethylnimodipine, 4-Hydroxy Nisoldipine,	3	MetLin	Siderophores	Down	− 7.0	0.614	0.003575	0.010449
15	8.21 min 299.260 m/z	[M−H]–	n/a	C18H36O3	2	Hydroxy octadecanoic acid, Hydroxy stearic acid	3	MetLin	Long-chain fatty acids	Down	− 4.0	0.572	0.012189	0.024379
18	8.23 min 281.249 m/z	[M−H]–	n/a	C18H34O2	1	Octadenoic acid	3	MetLin	Fatty acid	Down	− 3.0	0.52	0.4428	0.50989
19	8.18 min 635.527 m/z	[M−H]–	279.232	C43H72O3	22	1-(8-[3] -ladderane-octanyl)-2-(8-[3]-ladderane-octanyl)-sn-glycerol	2	MetFrag	Glycerolipids	Up	2.	0.52	0.34365	0.41485
20	7.93 min 279.203 m/z	[M−H]–	235.205	C17H28O3	23	10-Heptadecatrienoic acid	2	MetFrag	Receptor for protein	Down	− 1.0	0.52	0.72667	0.76704
22	3.15 min 346.059 m/z	[M−H]–	134.047, 211.007	C10H14N5O7P	9	Adenosine monophosphate	2	MetLin	Nucleotide	Down	− 7.0	0.48	0.008747	0.019553
23	3.94 min 335.076 m/z	[M−H]–	n/a	C16H16O8	3	3-Caffeoyl-1,5-quinolactone, 3-*O*-Caffeoylshikimic acid, 4-*O*-Caffeoylshikimic acid	3	MetLin	Cinnamic acids and derivatives	Down	− 5.0	0.41	0.000196	0.00124
24	2.35 min 214.048 m/z	[M−H]–	140.006, 84.558	C5H14NO6P	1	sn-Glycero-3-phosphoethanolamine	2	MetFrag	Glycerophospholipid	Down	− 2.0	0.38	0.14053	0.19779
25	3.00 min 306.078 m/z	[M−H]–	141.061, 128.023, 127.051, 143.045	C10H17N3O6S	4	Glutathione	2	MetLin	Coenzyme	Down	− 3.0	0.38	0.005326	0.014456
27	2.28 min 253.085 m/z	[M−H]–	209.060, 136.006	C16H14O3	7	Unknown	4	N.A	N.A	Up	2.0	0.31	0.22601	0.29615
28	3.23 min 133.014 m/z	[M−H]–	n/a	C4H6O5	1	Malic acid	3	MetLin	Organic acid	Down	− 3.0	0.27	0.036495	0.063038
29	2.34 min 471.151 m/z	[M−H]–	n/a	C21H28O12	0	1-*O*-Cinnamoyl-beta-d-gentiobiose	3	MetLin	Phenylpropanoid	Down	− 2.0	0.23	0.078267	0.11897
30	7.44 min 399.277 m/z	[M−H]–	n/a	C22H40O6	4	3-*O*-Cetyl ascorbic acid, Nonadecane-1,1,1-tricarboxylic acid	3	MetLin	Organic acid derivative	Down	-1.0	0.12	0.48306	0.53989

All metabolites were identified at fragmentation level (if fragments available). ^a^ level of identification; 1: the identification was verified with standard at fragmentation level, 2: the identification was done at fragmentation level by matching with online library as stated in reference column, 3: the identification was done using SmartFormula (Bruker, Germany) and mass accuracy solely based on parent ion mass due to unavailable fragments. ^b^ VIP: variable importance projector which indicate the variance of sample cause by the metabolite. VIP > 0.95 indicates the metabolite cause high variance among sample subject to cross refer with p-value and FDR (false discovery rate). * Significant different metabolites from VIP > 0.95 and supported with p-value < 0.01 and FDR cut-off 0.01

Unlike intracellular, untargeted profiling of extracellular metabolites of both MSM-G and MSM-P cultures have detected trehalose as the only molecule with above the limit of detection. This result indicates that *P. curvatum* is not active in secreting polar molecules outside the cells. Trehalose is a non-reducing sugar and an abundance compatible solute in fungal cells is known for its functions as stress protectant [22]. The finding suggests that trehalose is likely an important stress protectant molecule for *P. curvatum*, similar to *Xanthophyllomyces dendrorhous* strains that accumulate trehalose as stress protectant against reactive oxygen species [23].

To distinguish specific intracellular metabolites that significantly differ between MSM-P and MSM-G cultures, partial least squared-discriminant analysis (PLS-DA) was carried out. The PLS-DA plot shows the difference of MSP-P and MSM-G metabolome as explained by PLS 1 with 0.658 (Fig. 2b). Further analysis based on the VIP > 0.95, *p* > 0.01, and FDR cut-off 0.01 have identified seven 7 intracellular metabolites that have significant difference between MSM-P and MSM-G (Table 2). These metabolites include trehalose, glycerophosphocholine, sn-glycero-3-phospho-1-inositol, citric acid, and two unknown metabolites with less abundance in MSM-P culture. Only one unknown metabolite was found highly abundant in MSM-P culture.

### Changes in targeted organic acids concentration upon glucose substitution of palm oil in minimal media cultures

**Fig. 3:**
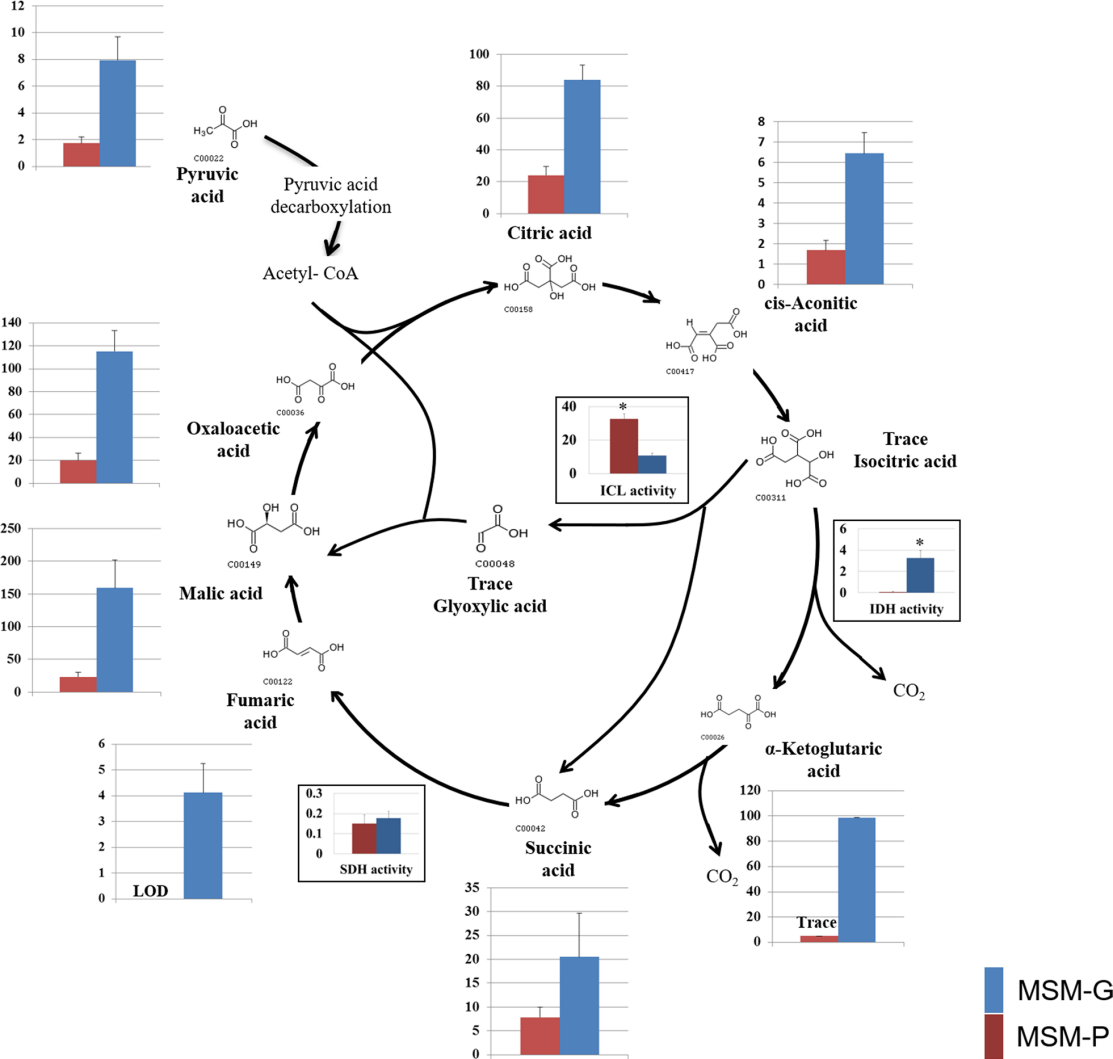
The organic acids and enzyme activities in TCA and glyoxylate cycle from intracellular extracts of *P. curvatum* AW02. Organic acid concentration was indicated with mg/g biomass and enzymatic activity was measured in U/g biomass. Asterisk indicated significant difference enzymatic activity with p < 0.05. LOD: limit of detection, Trace: limit of quantitation (LOQ). Details on the LOD and LOQ for each targeted organic acid was described in Additional file 1: Table S4

To understand the central carbon metabolism of an omnipotent fungus upon catabolising carbohydrates and triglycerides as sole carbon source, all organic acids involved in TCA and glyoxylate cycle were targeted and quantified from intracellular extracts of MSM-P and MSM-G cultures (Fig. 3) (Additional file 1: Table S2).

In MSM-G culture, the organic acids from TCA cycle were all detected and quantified. The results show that *P. curvatum* AW02 grown on glucose produced high malic acid (159 mg/g) compared to other TCA intermediates such as citric acid (84 mg/g) and succinic acid (21 mg/g). Ratio of organic acids composition in the TCA cycle of *P. curvatum* AW02 was found similar with *Y. lipolytica* that grown on glucose, in which high concentration of malic acid was observed, followed by fumaric acid and citric acid [24]. Nonetheless, this ratio of organic acids composition profile was shown to be different than *S. cerevisiae*. It was proposed that intracellular metabolite concentrations in TCA cycle is species-specific [14].

In MSM-P culture, only trace concentration of alpha-ketoglutaric acid was observed while the fumaric acid was under limit of detection. These showed that the TCA cycle for MSM-P that we observed was incomplete, suggesting that MSM-P culture recruit glyoxylate cycle as the key metabolic pathway at central carbon metabolism. This was supported by higher abundance of glyoxylic acid in MSM-P compared to the extract of MSM-G culture (Additional file 1: Fig. S4B). Furthermore, the citric acid, succinic acid, malic acid, and oxaloacetic acid were found at ~ 4- to 8-fold lower compared to the MSM-G culture. This observation is in agreement with the previous *Y. lipolytica* study that showed high concentration of malic acid, fumaric acid, succinic acid, and pyruvic acid in glucose-grown when compared to oleic acid-grown [24].

### Enzymatic assay of ICL, IDH, and SDH

To further understand the correlation of organic acids concentration and key enzymes involved in the activation of glyoxylate cycle upon growing on different carbon source, the activities of selected enzymes in TCA and glyoxylate cycle were determined by enzymatic assays. Isocitrate lyase (ICL) and isocitrate dehydrogenase (IDH) involved in regulating TCA and glyoxylate cycle were shown significantly up-regulated (3-fold) and down-regulated (−3-fold), respectively when palm oil was used as carbon source (Fig. 3). These results are in agreement with the low concentration of α-ketoglutaric acid observed in MSM-P culture. Up-regulation of ICL and down-regulation of IDH is known for the activation of glyoxylate cycle. ICL cleaves isocitric acid to glyoxylic acid and succinic acid, which limits the production of α-ketoglutaric. The low activity of IDH may be due to the phosphorylation of IDH, which could happen when the fungus grows under glucose starvation condition [25].

### Metabolic pathway analysis

The analysis was carried out to identify the changes in metabolic pathway of *P. curvatum* AWO2 grown on two different carbon sources. *Saccharomyces cerevisiae* metabolome (Kyoto Encyclopaedia of Genes and Genomes) was used as the model organism for metabolic pathway topology and enrichment analysis. Combinations of metabolites obtained from identification Level 1 and 2 of untargeted metabolite profiling (Table 2) and targeted organic acid (Additional file 1: Table S2) were used in the analysis.

**Fig. 4:**
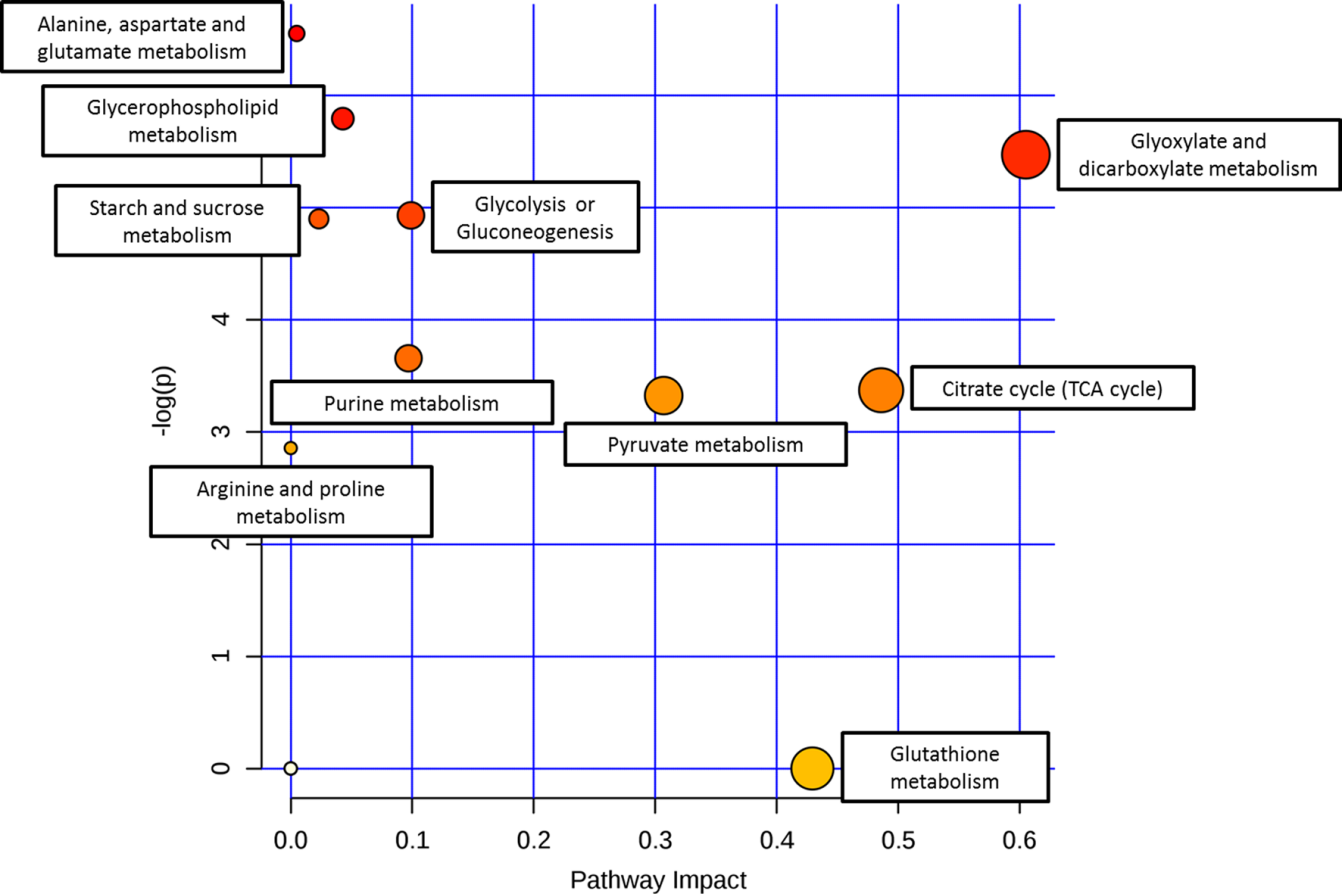
Summary of metabolic pathway analysis using MetaboAnalyst 3.0. The analysis is a combination of pathway enrichment analysis and pathway topology analysis. Pathway enrichment analysis provided the significant value of -log(p) (axis-y) for each pathway based on value of each metabolite. Pathway topology analysis gave pathway impact value (axis-x) based on the centrality of a metabolite within a pathway. Red colour represents the most significant regulated pathway while the size of spot represents pathway impact value

The results show that glyoxylate and dicarboxylate metabolism, tricarboxylic acid cycle (TCA cycle), and pyruvate metabolism were regulated differently when palm oil replaced glucose as carbon source (-log(p) > 3, pathway impact > 0.1, Fig. 4, Additional file 1: Table S3). This revealed that consumption of palm oil as carbon source results in metabolic changes at central carbon metabolism, which involves glyoxylate and dicarboxylate metabolism, TCA cycle, and pyruvate metabolism. Nonetheless, other metabolism like amino acids metabolism (alanine, aspartate, and glutamate metabolism), glycerophospholipid metabolism, glycolysis or gluconeogenesis, starch and sucrose metabolism, and purine metabolism were also significantly affected but with lower impact value.

## Discussion

### Proposed metabolic pathway network of *P. curvatum* AW02 grown on palm oil replacing glucose as carbon source

**Fig. 5:**
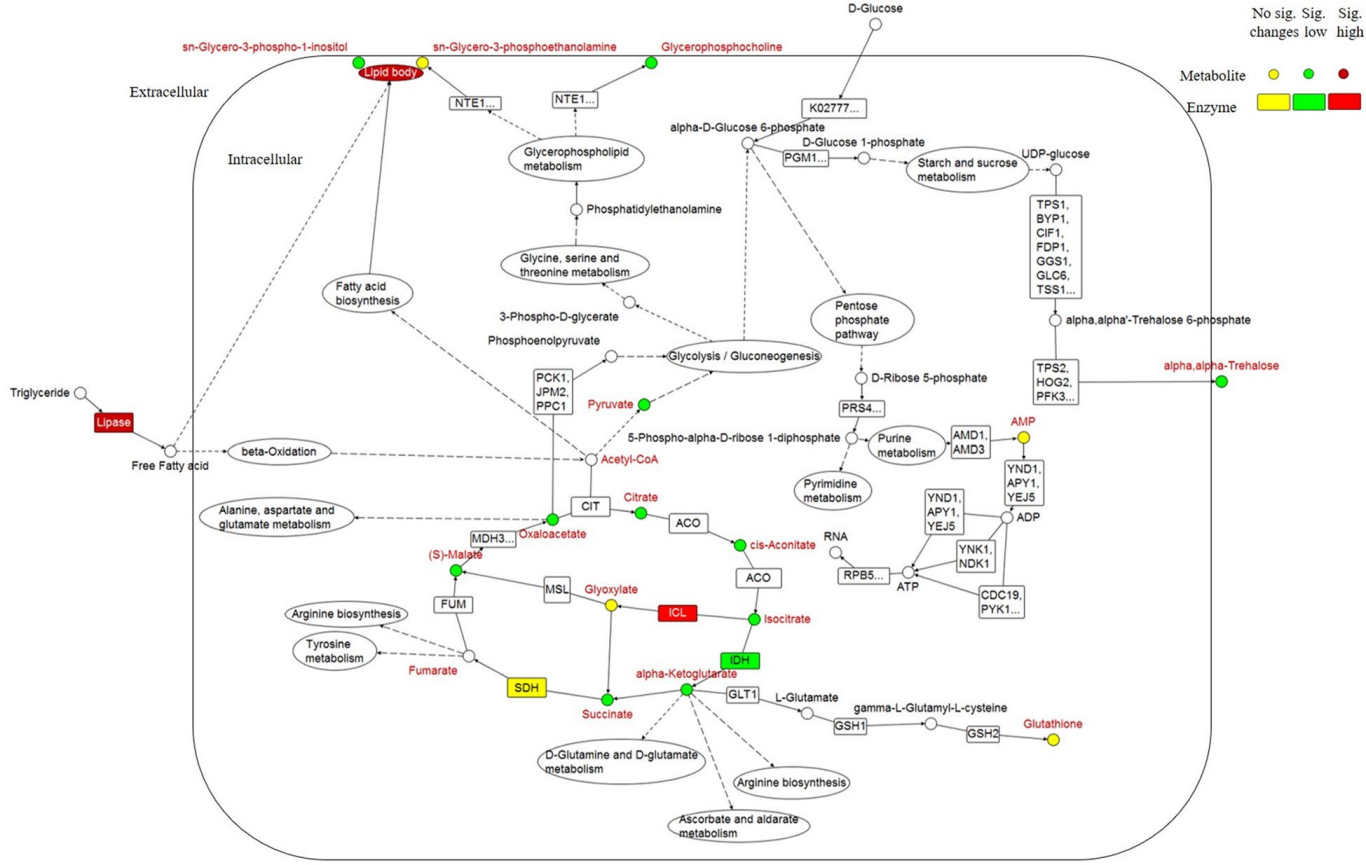
Overview of the metabolites, major metabolic pathways and pathway-related enzyme changes in palm oil-grown. The metabolites (circle) and enzymes (box, KEGG code) are shown in colour. The major metabolic pathways are presented in oval. Dashed lines indicate multiple hidden steps in the pathway

To obtain better insights on the possible metabolic pathway network of *P. curvatum* AW02 that utilised palm oil, data from enzymatic assay (lipase, IDH, ICL, and SDH), untargeted metabolite profiling, and targeted organic acid analysis were integrated using Vanted (Visualization and Analysis of Networks containing Experimental Data) software [26] (Fig. 5).

In MSM-P medium that contains palm oil as the sole carbon source, high activity of extracellular lipase was detected (Fig. 1D & 5). The recorded lipase activity of 300 U/100 mL in MSM-P was found, which is 2.8-fold higher as compared to previous study (34 – 170 U/100 mL), in which *P. curvatum* AW02 was cultivated on rapeseed oil as carbon source [6]. When glucose was the sole source of carbon and energy, lipase activity was found to be 10^−2^-fold lower. The mechanism of regulation occurs probably on the level of transcription as shown in other ascomycetes including *Beauveria bassiana* that recently reported [27] where deletion of the gene *Bbctf1β*, which encoding a zinc finger transcription factor, led to a significant reduction of the expression of nine lipase genes.

The lipase activity hydrolyses triglyceride from palm oil into glycerol and free fatty acids. Fatty acids are known to be transported into the intracellular compartment and are further degraded during β-oxidation process [21]. To grow on the obtained acetyl-CoA, isocitrate lyase will need to provide malate synthase with glyoxylate. The detected increase in isocitrate lyase activity is expected because ICL is known to be repressed by transcription factor *cre A* in *Aspergillus* species [28], which ortholog also identified as *mig 1* in *Saccharomyces cerevisiae* [29, 30]. This, regulation is well known as Carbon Catabolite Repression [31].

The concentration of organic acids in TCA/glyoxylate cycle was found to be 4- to 8-folds lower in palm oil-grown as compared to glucose-grown (Fig. 3 & 5). The low concentration of organic acids from fungi that grow on hydrophobic substrate such as vegetable oil might due to the accumulation of lipid [24, 32]. In agreement to this, this study observed abundance of lipid bodies in the intracellular compartment of palm oil-grown when compared to the glucose-grown.

This study also revealed that palm oil substitution affects the metabolisms that are highly dependent on carbon metabolism. For instance, trehalose concentration in palm oil-grown mycelium was found to be 4-fold lower compared to glucose-grown culture (Additional file 1: Table 2, Additional file 1: Fig. S5). Two explanations are possible: (i) Due to the limited supply of glucose, biosynthesis of trehalose have to run via gluconeogenesis after catabolism of triglyceride in palm oil-grown whereas glucose-grown can simply take up the monomer and convert it into trehalose, (ii) Since osmostress is higher in glucose-grown hyphae, more trehalose is needed and synthesised by glucose-grown in comparison with a medium containing the weakly soluble lipid. The function of trehalose as the compatible solute in fungi is well-known [33]. Trehalose protects proteins and membranes from the osmotic dehydration [34]. A previous study showed that an increase of trehalose concentration in both, intra- and extracellular room, was due to the increased osmotic pressure on *Corynebacterium glutamicum* [35]. To reveal the possible role of trehalose that was 4-fold higher in MSM-G grown media, the osmolality of MSM-G and MSM-P was measured. The results showed that MSM-G medium indeed had higher osmolality (91 ± 6 mOsm/kg) than MSM-P medium (71.8 ± 7 mOsm/kg). The osmolality of MSM-G was then significantly reduced after 5-days cultivation most probably caused by an up-take of glucose by *P. curvatum* AW02 (Additional file 1: Table S5 & Fig. S6) and followed by the increase of trehalose concentration. Note that, the MSM-G and MSM-P media after 5-days cultivation reached a similar osmolality level. Since osmoregulation works fast the remains of trehalose appear as an anabolic memory.

On the other hand, the metabolism that is less influenced by carbon metabolism were found not to be affected by palm oil substitute glucose as carbon source. For instance, glutathione concentration was found similar in both palm-oil grown and glucose-grown biomass extracts (Table 2, Additional file 1: Fig. S5). Previous study showed that glutathione was highly affected by the source of nitrogen [36]. Thus, the replacement of glucose with palm oil as carbon source did not affect glutathione biosynthesis since both cultures were supplemented with potassium nitrate as nitrogen source.

This study identified sn-glycero-3-phospho-1-inositol and sn-glycero-3-phosphoethanolamine, a candidate of glycerophospholipids that may play a role in the lipid bodies formation of *P. curvatum* AW02 (Fig. 5). It is known that oleaginous fungi commonly form a monolayer of phospholipids coating intracellular lipid bodies [37]. Hence, this study hypothesised that the identified glycerophospholipids may have contributed to the formation of lipid bodies, which was visualized in palm oil-grown hyphae (Fig. 1E & F). Furthermore, this molecule had been reported to correlate with membrane integrity during adaptation of *Candida glabrata* towards osmotic stress [38]. The level of membrane integrity was also proposed to be correlated with the co-existence of fatty acid such as palmitoleic acid, as has been shown in *S. cerevisiae* [39, 40]. Note that, palmitoleic acid is one of the fatty acids found in palm oil [41]. Taken all together, this study hypothesised that the glycerophospholipids may have taken part in: (i) Lipid bodies formation and (ii) Membrane cells integration, which cause the glycerophospholipids to be less abundant in the palm oil-grown intracellular sample.

## Conclusions

In summary, this study investigated the metabolite and central carbon metabolism changes of *P. curvatum* AW02 based on the mineral salts medium with different carbon sources; palm oil versus glucose. The replacement of glucose with palm oil was shown to induce physiology changes in *P. curvatum* AW02 including longer lag phase, lipase activity, and contain abundance of elongated lipid bodies in the hyphae. Comparative metabolomics reveals significant changes at the central carbon metabolism involving glyoxylate and TCA cycles as well as pathways that are related to the carbon metabolism. As expected, an activation of glyoxylate pathway was shown in lipid-degrading hyphae through the detection of higher abundance of glyoxylic acid and up-regulation of isocitrate lyase activity. Concerning trehalose, lower contents in the extracts of palm oil-grown hyphae were expected and found. Trehalose, a stress response metabolite indicated less stress in palm oil-grown; i.e. due to its reduced osmotic activity. The strong staining response of intracellular lipid suggested that it is the reserve. This study suggested that palm oil is a potential alternative carbon source for a minimal sterilised fermentation process. More importantly, the results showed that the concentration among each organic acid in the TCA and glyocylic pathway varied significantly. As malic and oxaloacetic acids concentration were found higher in glucose-grown cultures, citric acid was found the most abundance at palm oil-grown conditions. This provides evidence that central carbon metabolism of the omnipotent filamentous fungus *P. curvatum* undergone significant changes in response towards the triglyceride and sugar-based carbon source. It also suggests that a carbon flow optimisation may have taken place in the cells during palm oil as carbon source. This observation is important for the development of strategies in fermentation processes that can be implemented on organic acids production or secondary metabolite such as 4-hydroxybenzoic acid.

## Methods

### Fungus

*P. curvatum* AW02 (DSM 23903) was pre-cultured on rich media HA agar prior to culture in mineral salt media (MSM) with different carbon sources. Cultivations were carried out in 500 mL Duran baffled flasks (Schott, Germany) containing 100 mL medium at 33 °C with agitation of 120 rpm [6].

### Medium composition

Rich media (HA) contained 10 g/L yeast extract (YE), 10 g/L glucose, and 20 g/L agar. Modified mineral salt media (MSM) contained 1.5 g/L KNO_3_, 0.5 g/L MgSO_4_·7H_2_O, 0.5 mg/L FeSO_4_·7H_2_O, 0.5 mg/L ZnSO_4_·7H_2_0, 0.02 mg/L CuSO_4_·5H_2_O, 0.02/L mg MnCl_2_·4H_2_O, and 1.5 g/L KH_2_PO_3_. MSM was added with 18 mL of crude palm oil/L (CPO) as MSM-P or added with 10 g/L glucose as MSM-G. No growth factors including vitamins were included. To prepare MSM-P or MSM-G agar plate, 20 g of bacteriological agar was applied per litre media. All media was adjusted to pH 3 using HCl [6].

### Colony growth rate determination for *P. curvatum* AW02

The growth rate of *P. curvatum* AW02 in HA medium (control), MSM-P, and MSM-G was determined according to [6]. In short, 10 µL of glycerol stock was inoculated on HA, MSM-P, and MSM-G agar. The experiment was prepared in triplicate. Duplicated experiment was performed. Visible colony diameter was measured on daily basis. The growth rate, K_r_ was determined by calculating slope of exponential phase, where K_r_ = (R_1_-R_0_)/(t_1_-t_0_) [42, 43] which R_0_ and R_1_ represent the colony diameter at time t_0_ and t_1_, respectively.

### Biomass production

About 10 µL of mycelia glycerol stock of *P. curvatum* AW02 previously cultured in HA broth for 5 days was inoculated on HA agar and incubated at 30 °C for minimum 3 days as described previously [6]. Young mycelia (the outer colony) were collected and weighted before transferred into 50 mL falcon tube that contained 30 glass beads (5 mm diameter) and 10 mL of 0.9% (w/v) NaCl. The sample was vortexed until fine dispersed mycelia suspension was obtained. The final inoculums at concentration of 2 mg/mL was prepared.

Two mL of *P. curvatum* AW02 inoculums (2% v/v) was inoculated into baffle flask (500 mL) that contained 100 mL of MSM-P or MSM-G broth. The cultures were incubated for 12 days. Sample was harvested for every 2 days. The experiment was prepared in triplicate. To harvest the biomass, two steps filtration was applied. Firstly, the excess palm oil that may still stick on mycelium with organic solvent ethyl acetate was removed. Secondly, the biomass was obtained (data not published). In short, the culture was filtered through a 0.2 µm nylon filter membrane (Sartorius Stedim, Germany). The retained mycelium was then mixed with 1:1 of ethyl acetate by vigorous shaking. The mixture was rested ~ 10 min until clear separation between organic and aqueous phase was obtained. Later, the organic phase that contained crude palm oil was removed and the aqueous phase that contains mycelium was filtered through a 0.2 µm nylon filter paper (Sartorius Stedim, Germany). The retained mycelium was dried overnight in incubator oven at 70 °C before weighted as dry biomass.

### Lipase assay

*P. curvatum* AW02 that cultured in MSM-P and MSM-G were harvested and centrifuged at 6000 × *g* for 6 min at room temperature. To detect the extracellular lipase of *P. curvatum* AW02, 100 μL supernatant of MSM-P or MSM-G culture were used to incubate with 900 μL substrate containing pNPP (p-nitrophenylpalmitate) for 4 min at room temperature to measure lipase activity. The substrate was freshly prepared by mixing solution A (30 mg of pNPP dissolved in 10 mL isopropanol) and solution B (0.8 mg of Triton X-100 and 0.1 mg gum arabicum that dissolved in 100 mL 0.1 M Tris–HCl, pH 8) in a ratio of 1: 9. The lipase activity was measured according to the release of p-nitrophenol from pNPP upon hydrolysis process by extracellular lipase through spectrophotometer absorbance at 405 nm. One unit (U) was defined as the amount of lipase needed to liberate 1 µmol p-nitrophenol per minute under the previous described conditions [6].

### Lipid bodies visualization

Lipid bodies visualization was carried out to observe the possible changes in lipid bodies presence in *P. curvatum* AWO2 cells grew on palm oil as compared to glucose as carbon source. About 100 µL of *P. curvatum* AW02 that cultured in MSM-P and MSM-G broth were mixed with 10 mL Nile red solution (1 mg/mL acetone). The samples were mounted on a glass slide and viewed using Zeiss fluorescence microscope (ZEISS, Germany) with the filter combination BP 375-425/FT 425/LP. Lipid bodies were visualised as intense yellow fluorescence [20, 44].

### Total cellular lipid

Lipid was extracted out from intracellular compartment by using chloroform–methanol method [45]. Briefly, samples from MSM-P and MSM-G cultures were harvested to obtain biomass. The biomass was freeze dried and suspended in mixture of chloroform/methanol solvent (2:1), before vortexed with glass beads for 20 min. Then, the organic phase was washed with 0.4 mL of 0.9% NaCl (w/v) before being dried at 60 °C for overnight. The total cellular lipid content was expressed as gram of lipid per gram of biomass (%).

### Enzymatic assay of isocitrate lyase, isocitrate dehydrogenase, and succinate dehydrogenase assay

The enzymatic activity of isocitrate lyase was assayed using ICL assay kit (MyBioSource, USA) as manufacturer’s instructions. One unit of ICL activity is defined when the enzyme decomposes of 1 µmol of the NADH per minute. Meanwhile, the level of isocitrate dehydrogenase was determined using IDH activity assay kit (Sigma-Aldrich, Malaysia) as per manufacturer’s instructions. One unit of IDH is the amount of enzyme that will generate one μmol of NADH or NADP per minute at pH of 8.0 and at 37 °C. The activity of succinate dehydrogenase was determined by a colorimetric method using succinate dehydrogenase activity colorimetric assay kit (BioVision, USA). One unit of SDH is the amount of enzyme that generates 1 µmol of dichlorophenolindophenol (DCIP) per minute at pH of 7.2 and at 25 °C.

All reactions were carried out in microtiter plates and scanned at each respective wavelength using microtiter plate reader (Tecan, Switzerland).

### Determination of osmolality

The osmolality of the medium MSM-G and MSM-P were analysed by using a Micro-Osmometer Model 3320 (Advanced Instruments Inc., USA) [46, 47]. Briefly, 20 μL samples of medium before cultivation and after 5 days cultivation were analysed in triplicates using the osmometer with distilled water as a reference. The results were given as mOsm/kg.

### Metabolomics analysis

To perform metabolomics analysis of *P. curvatum* AW02 samples, instruments including LC–MS-TOF/LC–MS/MS-QTOF, LC–MS/MS-TripleQ, and GC–MS were employed, similar to previous study by [48] and the workflow is summarised in Additional file 1: Fig. S7.

#### Metabolites extraction

The following steps were carried out as described previously [49, 50]. One volume of sample culture was mixed with 5 volumes of quenching solution (60% methanol, 10 mM HEPES, pH 7.5, −40 °C). Later, the mixture was kept at −40 °C for 3 – 5 min. The mixtures were centrifuged (5000 *g*, 6 min at 0 °C) to separate pellet/mycelia (intracellular) and supernatant (extracellular). The extracellular sample was used directly for metabolite analysis. The intracellular metabolites sample from mycelia was extracted using 5 mL extraction solution (75% ethanol, 10 mM HEPES, pH 7.5) at 80 °C and incubated for 5 min. Then, the lysate was chilled in ice for 5 min and subsequently centrifuged for 5000 *g* at 4 °C for 10 min. The supernatants of cell lysate or extract of polar and semi-polar metabolites were used for the analysis. Finally, camphorsulfonic acid and gallic acid were spiked into the cell lysate as internal standard with the final concentrations of 10 ppm.

#### LC–MS-TOF condition and data acquisition

Sample extracts from previous section were analysed using UPLC-ESI–MS Micro Time of Flight (Bruker, Germany). Samples were separated with reverse phase chromatography using C18 column at 45 °C. The mobile phase was water with 0.1% formic acid (A) and acetonitrile (B). The gradient flow of mobile phase composition for liquid chromatography (LC) was programmed as follows: 5 to 40% B (v/v) in 3 min, then to 95% B (v/v) until 5 min and hold for 10 s, sharply decreased to 5% B (v/v) and maintained until 15 min. The flow rate was 0.3 mL/minute and injection volume was 3 μL [51].

In mass spectrometry (MS), mass spectra were generated by electrospray ionization (ESI) in negative mode with a range of m/z 50 to 1000 for scanning. The acquisition parameters were as follows: 4 kV of capillary voltage, 8 L/minute of dry gas, 200 °C of dry gas heater, nebulizer at 1.2 bar. The calibration of MS was done in every sample injection using 180 μL/hour of a sodium formate cluster mix which contained minimum 7 calibration points with mass range 112.9856 – 996.8221 m/z to check mass precision in each run.

#### Data processing, multivariate, and statistical analyses

The mass spectra data were processed using Compass software of DataAnalysis and ProfileAnalysis (Bruker, Germany), which served to align the recorded m/z and retention from each sample in a bucket form. Find molecular features (FMF) algorithm was used by performing the advanced bucketing, where each bucket will represent metabolites and contains intensity value [52]. The data matrix that contain the list of all buckets or metabolites along with their intensity values were generated and exported to metaboanalyst online programme (http://www.metaboanalyst.ca) for normalisation of intensities and statistical analysis. Normalisation was done based on spikes with an internal standard in each sample.

For statistical analysis, Student’s *t* test was performed using both MSM-P and MSM-G cultures, treated as paired sample. The significant changes in metabolites between these culture set were identified at *p* < 0.01 and false discovery rate (FDR) cut-off at 0.01.

SIMCA 14.1 (Umetrics, Sweden) was used for multivariate analysis. The normalised data matrix was scaled using Pareto before analysed using principal component analysis (PCA) and partial least squares discriminant analysis (PLS-DA). Pareto scaling was used to overcome the problem of strong variations of intensity between different metabolites. To identify the metabolites that contribute to metabolites variation, they were arranged according to their importance in projecting the variations. Metabolites with variable importance in the projection (VIP) values more than 0.95 were identified as the metabolites that significantly changes between MSM-P and MSM-G cultures [53].

#### Method validation, quality control, and data treatment

Internal standard of gallic acid was used to demonstrate precision, stability, and repeatability of the method according to the variation of peak intensities and retention times. The average recorded retention time and m/z were 5.5 min: 169.013 m/z in negative ion mode. The relative standard deviations (RSDs) of peak intensities and retention times (RT) were estimated to be 6 and 0%, respectively.

Pooled sample of MSM-P and MSM-G culture were prepared for each set of intracellular and extracellular and treated as quality control (QC). Later, the generated data matrix from ProfileAnalysis (Bruker, Germany) software were filtered by cross-checking each sample of MSM-P or MSM-G culture with QC sample to ensure a good repeatable analysis [54].

#### LC–MS/MS-QTOF and metabolite identification

Automated fragmentation had been applied for all acquit precursor ion mass in negative mode using UPLC-ESI–MS/MS Micro Quadrupole-Time of Flight (Bruker, Germany). The metabolites were tentatively identified at fragmentation level. The mass of precursor ion during MS were corroborated with their product ion during MS/MS fragmentation. Later, the information was used to identify the metabolite based on fragments hit and neutral loss using online database, METLIN (https://metlin.scripps.edu) and MassBank (http://www.massbank.jp). The neutral loss calculation was performed according to loss of functional group such as carboxyl during fragmentation or ion adduct that react to precursor ion during ionization process [55]. In-silico fragmentation using MetFrag (https://msbi.ipb-halle.de/MetFragBeta/) was applied when both fragments hit and neutral loss provided no information from online database.

The level of identification for metabolites identified in this study was ranged based on criteria described previously [56] with some modifications. Identification Level 1 was assigned for metabolites that validated with authentic standard, whereas Level 2 was for tentatively identified metabolites that could not be validated by authentic standard. In the case of daughter ions that were not unavailable, parent ions were used for the identification and assigned as Level 3. The identification at this level was stated as tentatively characterised class. Identification Level 4 applied for unknown metabolites.

#### UPLC-ESI–MS/MS TripleQ condition and data acquisition for targeting organic acids

Targeted organic acids in intracellular of MSM-P and MSM-G culture were quantified using UPLC-ESI–MS/MS Triple quadropole (Waters, USA). The intracellular metabolites samples from metabolite extraction section were used and separated using Acquity UPLC HSS T3 1.8 µm, C18 column at temperature of 45 °C. The mobile phase, gradient flow, and mass spectra acquisition parameter were same as LC–MS-TOF as described previously.

#### Organic acids quantification

Multiple reactions monitoring (MRM) was used to target organic acid in intracellular samples of MSM-P and MSM-G culture along with their daughter ions during fragmentation. Collision energy and optimal depolarization potentials of targeted organic acids was first determined by injecting organic acids standards into the mass spectrometer (Table 3). The chromatogram of each organic acid standard with their respective RT is shown in Additional file 1: Fig. S8.

**Table 3:** Organic acid standards and parameter accusation

Organic acid	RT (min)	MRM (Parent > Daughter ion)	Collision energy (eV)
Pyruvic acid	1.01	87 > 43	10
Fumaric acid	1.42	114.9 > 7.09	20
Oxaloacetic acid	1.01	132.9 > 87	20
Malic acid	1.01	133.3 > 71	14
		133.3 > 114.8	
alpha-Ketoglutaric acid	1.08	114.9 > 57	12
		114.9 > 101	12
cis-Aconitic acid	1.27	172.8 > 84.9	12
Citric acid	1.21	190.8 > 87	18
		190.8 > 110.9	12
Iso-citric acid	1.01	191.9 > 110.9	20
		191.9 > 72.10	14
Succinic acid	1.48	116.7 > 73.4	20
Camphorsulfonic acid	2.65	231.2 > 80.04	32

The concentration of targeted organic acids was determined according to the standard curve of organic acids standards with concentration of 0.2, 0.4, 0.6, 0.8, 1.0, 2, 4, 6, 8, and 10 ppm using TargetLynx™ software (Waters, USA).

#### Detection and quantification of glyoxylic acid via GC–MS

The intracellular metabolites samples from metabolite extraction section were incubated with 50 µL methoxyamine solution (25 mg/mL methoxyamine hydrochloride in pyridine) for 30 min at 60 °C. Subsequently, 50 µL of TMS reagent (BSTFA/TMCS, 99:1) was added into the mixture and incubated for 60 min at 60 °C. In this derivatization step, the keto group of glyoxylic acid will be methoxylated into methoxyamino groups, whereas hydroxyl group will be added with trimethysilyl during trimethysilation [48].

Glyoxylic acid detection and quantification was performed using GC–MS (Agilent, USA) coupled with BP-20 polar column (30 m × 250 μm, 0.25 μm film thickness). Helium gas was used as carrier at 1.3 mL/min. One μL sample was injected in split-less mode via autosampler. The inlet temperature was set at 280 °C. The temperature program of oven was started with 40 °C for 3 min and gradually increased with the rate of 5 °C/min for 36 min until the temperature achieved 220 °C, and maintained for 3 min. Mass spectra were collected with the range of 45 to 600 m/z with 7 min solvent delay.

Single ion monitoring (SIM) mode was used to detect glyoxylic acid from sample. Initially, glyoxylic acid standard was analysed through GC–MS using scan mode to obtain mass spectra. The gathered mass spectra (Additional file 1: Fig. S4A) was cross checked with previous study [48] and used as reference in SIM mode to detect glyoxylic acid from sample.

Standard curve was built according to glyoxylic acids standards measured at various concentration (62.5, 100, 125, 500, and 1000 ppm). Later, the quantification of glyoxylic acid in sample was based on the developed standard curve.

### Statistical analysis

Student’s *t*-test was performed in lipase and enzymatic assay to verify the differences between MSM-P and MSM-G cultures from the obtained data. Each experiment was repeated at least 2 times with each experiment run in triplicate. The probability was set at *p* < 0.05. Statistical analyses were performed using the software Statistical Package for the Social Sciences (SPSS) 18.0 (IBM, USA).

## Supplementary information

**Additional file 1.** Additional tables and figures.

## Data Availability

All data generated or analysed during this study are included in this published article and its additional files.

## Abbreviations

MSM-P: Minimal medium with palm oil as carbon source
MSM-G: Minimal medium with glucose as carbon source
PCA: Principal component analysis
PLS-DA: Partial least square discriminate analysis
VIP: Variable importance in projection
ICL: Isocitrate lyase
IDH: Isocitrate dehydrogenase
SDH: Succinate dehydrogenase
QC: Quality control
