# Supplementary material for: Comparative metabolomics of Phialemonium curvatum as an omnipotent fungus cultivated on crude palm oil versus glucose
Source: Microb Cell Fact. 2020 Sep 9;19:179. doi: 10.1186/s12934-020-01434-w (PMC7487481; doi:10.1186/s12934-020-01434-w)
Supplement: Supplementary file 1 — Additional file 1. Additional tables and figures. [file 12934_2020_1434_MOESM1_ESM.docx]

**Additional File 1**

Comparative metabolomics of *Phialemonium curvatum* as an omnipotent fungus cultivated on crude palm oil *versus* glucose

Arief Izzairy Zamani^1^, Susann Barig^2^, Sarah Ibrahim^1^, Hirzun Mohd. Yusof^3^, Julia Ibrahim^3^, Jaime Yoke Sum Low^3^, Kua Shwu Fun^3^, Syarul Nataqain Baharum^1*^, Klaus-Peter Stahmann^2*^ & Chyan Leong Ng^1*^

^1^Institute of Systems Biology, Universiti Kebangsaan Malaysia (UKM), 43600 UKM, Bangi, Selangor, Malaysia,

^2^Insitute of Biotechnology, Brandenburg University of Technology Cottbus –Senftenberg, Universitaetsplatz 1, 01968 Senftenberg, Germany

^3^Sime Darby Technology Centre, 1st Floor Block B, UPM-MTDC Technology Centre III, Lebuh Silikon, UPM 43400 Serdang, Selangor

*Corresponding author

S.N.B: E-mail: nataqain@ukm.edu.my. Tel: +603 8921 4550

K-P.S: E-mail: Klaus-Peter.Stahmann@b-tu.de. Tel: +49-3573-85-913 / 801

C.L.N: E-mail: clng@ukm.edu.my. Tel: + 603 8921 4561

**Table S1** **List of total buckets (metabolites) identified from both culture base on Find molecular Features (FMF) algorithm.**

| Name | Mean (SD) of MSM-P | Mean (SD) of MSM-G |
| --- | --- | --- |
| 0.82min 606.078m/z | 15.888 (0.516) | 46.471 (53.205) |
| 1.65min 174.956m/z | 15.888 (0.516) | 28.948 (41.747) |
| 1.87min 180.970m/z | 25.047 (31.782) | 17.762 (4.167) |
| 1.87min 996.123m/z | 18.279 (8.220) | 17.762 (4.167) |
| 1.87min 996.831m/z | 25.053 (31.804) | 17.762 (4.167) |
| 1.88min 740.850m/z | 15.888 (0.516) | 35.179 (60.055) |
| 1.88min 860.852m/z | 42.190 (91.165) | 35.957 (62.106) |
| 1.89min 248.960m/z | 103.883 (159.500) | 43.601 (88.547) |
| 1.89min 536.887m/z | 15.888 (0.516) | 25.229 (25.771) |
| 1.89min 758.867m/z | 42.099 (90.721) | 17.762 (4.167) |
| 1.89min 792.864m/z | 15.888 (0.516) | 94.179 (194.533) |
| 1.89min 928.831m/z | 46.195 (104.915) | 17.762 (4.167) |
| 1.89min 588.899m/z | 155.847 (253.815) | 200.805 (225.048) |
| 1.89min 902.830m/z | 15.888 (0.516) | 24.340 (22.418) |
| 1.89min 725.377m/z | 15.888 (0.516) | 24.719 (25.970) |
| 1.89min 316.947m/z | 154.802 (205.409) | 45.897 (96.493) |
| 1.90min 452.922m/z | 220.709 (253.626) | 140.435 (229.065) |
| 1.90min 876.823m/z | 21.011 (18.006) | 28.999 (38.728) |
| 1.90min 808.834m/z | 22.757 (23.490) | 17.762 (4.167) |
| 1.90min 672.857m/z | 15.888 (0.516) | 24.708 (23.670) |
| 1.90min 604.877m/z | 38.882 (56.848) | 46.404 (52.270) |
| 1.99min 193.064m/z | 15.888 (0.516) | 19.725 (7.309) |
| 2.25min 729.285m/z | 20.430 (16.014) | 23.884 (21.183) |
| 2.26min 491.186m/z | 39.518 (42.759) | 54.531 (70.032) |
| 2.27min 383.162m/z | 15.888 (0.516) | 81.174 (64.368) |
| 2.28min 253.085m/z | 123.189 (80.135) | 32.072 (48.871) |
| 2.34min 471.151m/z | 25.536 (24.867) | 28.936 (38.318) |
| 2.34min 660.256m/z | 15.888 (0.516) | 32.302 (25.441) |
| 2.34min 807.313m/z | 15.888 (0.516) | 19.606 (7.264) |
| 2.34min 146.045m/z | 54.925 (62.242) | 340.642 (310.403) |
| 2.35min 214.048m/z | 76.233 (92.850) | 104.773 (138.429) |
| 2.35min 403.152m/z | 25.400 (22.168) | 141.143 (146.734) |
| 2.37min 559.208m/z | 15.888 (0.516) | 44.480 (48.493) |
| 2.37min 346.095m/z | 15.888 (0.516) | 26.257 (29.290) |
| 2.37min 302.101m/z | 281.754 (247.522) | 452.046 (249.451) |
| 2.38min 242.080m/z | 21.379 (19.279) | 37.516 (45.588) |
| 2.40min 484.181m/z | 22.682 (23.536) | 28.283 (24.276) |
| 2.41min 644.217m/z | 19.412 (8.231) | 54.987 (69.119) |
| 2.43min 419.170m/z | 70.689 (43.286) | 17.762 (4.167) |
| 2.45min 227.077m/z | 247.940 (207.492) | 362.309 (242.783) |
| 2.45min 181.071m/z | 775.089 (592.144) | 959.464 (633.915) |
| 2.47min 385.102m/z | 15.888 (0.516) | 22.698 (17.005) |
| 2.47min 771.210m/z | 15.888 (0.516) | 22.266 (15.667) |
| 2.47min 725.207m/z | 15.888 (0.516) | 30.930 (45.190) |
| 2.47min 729.233m/z | 198.854 (264.759) | 1937.688 (1305.354) |
| 2.48min 387.115m/z | 1452.425 (661.745) | 3351.128 (668.775) |
| 2.48min 728.734m/z | 15.888 (0.516) | 23.230 (18.785) |
| 2.49min 431.106m/z | 57.433 (54.066) | 177.940 (124.153) |
| 2.49min 341.110m/z | 15.888 (0.516) | 58.285 (48.349) |
| 2.60min 251.074m/z | 15.888 (0.516) | 23.725 (20.473) |
| 2.65min 586.158m/z | 15.888 (0.516) | 31.841 (32.792) |
| 2.70min 357.105m/z | 15.888 (0.516) | 25.201 (25.683) |
| 2.70min 237.091m/z | 26.273 (36.027) | 17.762 (4.167) |
| 2.80min 267.074m/z | 15.888 (0.516) | 29.490 (26.804) |
| 3.00min 306.078m/z | 15.888 (0.516) | 43.640 (59.947) |
| 3.07min 611.149m/z | 15.888 (0.516) | 58.565 (59.124) |
| 3.15min 346.059m/z | 15.888 (0.516) | 87.490 (102.969) |
| 3.23min 133.014m/z | 15.888 (0.516) | 29.429 (40.206) |
| 3.80min 333.061m/z | 56.083 (73.768) | 373.139 (258.873) |
| 3.82min 259.024m/z | 15.888 (0.516) | 85.579 (82.596) |
| 3.84min 128.035m/z | 15.888 (0.516) | 101.895 (106.345) |
| 3.94min 335.076m/z | 15.888 (0.516) | 64.134 (48.873) |
| 4.05min 191.020m/z | 69.264 (96.816) | 377.456 (269.885) |
| 5.50min 815.243m/z | 15.888 (0.516) | 37.489 (35.090) |
| 6.70min 610.415m/z | 15.888 (0.516) | 26.653 (33.822) |
| 6.73min 723.506m/z | 126.301 (135.029) | 396.026 (563.315) |
| 6.78min 949.674m/z | 29.256 (31.347) | 83.974 (136.285) |
| 6.86min 313.240m/z | 15.888 (0.516) | 97.075 (149.474) |
| 7.04min 459.203m/z | 35.077 (34.700) | 17.762 (4.167) |
| 7.08min 401.291m/z | 124.515 (189.913) | 29.253 (39.600) |
| 7.10min 297.245m/z | 53.583 (88.165) | 230.569 (231.871) |
| 7.10min 187.097m/z | 21.661 (19.928) | 17.762 (4.167) |
| 7.18min 279.233m/z | 26.638 (37.315) | 58.933 (98.700) |
| 7.28min 269.214m/z | 15.888 (0.516) | 29.141 (39.203) |
| 7.37min 313.239m/z | 15.888 (0.516) | 83.959 (124.928) |
| 7.44min 399.277m/z | 30.501 (50.643) | 17.762 (4.167) |
| 7.45min 345.230m/z | 58.658 (102.460) | 17.762 (4.167) |
| 7.47min 718.574m/z | 15.888 (0.516) | 30.518 (46.147) |
| 7.48min 315.254m/z | 240.382 (167.989) | 374.593 (662.921) |
| 7.69min 905.563m/z | 15.888 (0.516) | 83.130 (229.353) |
| 7.69min 452.280m/z | 269.802 (241.466) | 575.540 (441.418) |
| 7.70min 585.507m/z | 41.274 (46.987) | 17.762 (4.167) |
| 7.72min 635.522m/z | 26.506 (36.803) | 17.762 (4.167) |
| 7.74min 611.526m/z | 103.235 (117.917) | 17.762 (4.167) |
| 7.74min 271.229m/z | 318.505 (444.886) | 855.733 (805.076) |
| 7.76min 794.574m/z | 15.888 (0.516) | 37.569 (45.677) |
| 7.77min 674.545m/z | 15.888 (0.516) | 49.880 (57.323) |
| 7.79min 639.557m/z | 38.283 (58.865) | 17.762 (4.167) |
| 7.80min 285.209m/z | 15.888 (0.516) | 46.069 (99.714) |
| 7.83min 281.249m/z | 830.456 (1025.952) | 583.707 (1325.693) |
| 7.84min 255.233m/z | 384.916 (450.290) | 204.781 (466.645) |
| 7.85min 609.503m/z | 30.494 (34.832) | 17.762 (4.167) |
| 7.86min 327.243m/z | 111.174 (98.734) | 75.922 (111.714) |
| 7.88min 403.307m/z | 15.888 (0.516) | 26.266 (29.319) |
| 7.89min 561.489m/z | 78.985 (218.619) | 17.762 (4.167) |
| 7.90min 375.253m/z | 51.900 (66.695) | 73.101 (93.513) |
| 7.91min 563.506m/z | 326.418 (389.840) | 341.636 (594.635) |
| 7.91min 537.490m/z | 283.463 (316.336) | 244.749 (427.932) |
| 7.92min 511.475m/z | 109.914 (146.048) | 80.634 (149.471) |
| 7.93min 279.203m/z | 246.551 (469.057) | 179.815 (244.469) |
| 7.93min 379.238m/z | 15.888 (0.516) | 27.801 (34.410) |
| 7.94min 852.568m/z | 21.468 (19.409) | 17.762 (4.167) |
| 7.95min 550.322m/z | 40.298 (57.565) | 17.762 (4.167) |
| 7.97min 732.541m/z | 37.202 (55.709) | 17.762 (4.167) |
| 7.97min 329.234m/z | 390.920 (709.406) | 17.762 (4.167) |
| 7.98min 633.511m/z | 15.888 (0.516) | 22.821 (17.272) |
| 7.99min 659.483m/z | 22.083 (21.393) | 17.762 (4.167) |
| 8.00min 301.238m/z | 26.002 (35.059) | 17.762 (4.167) |
| 8.01min 295.228m/z | 69.324 (80.724) | 240.880 (246.642) |
| 8.01min 277.214m/z | 15.888 (0.516) | 24.573 (25.469) |
| 8.01min 529.284m/z | 40.074 (46.206) | 117.721 (95.229) |
| 8.02min 399.277m/z | 100.197 (154.873) | 359.126 (249.538) |
| 8.07min 591.466m/z | 23.110 (24.950) | 17.762 (4.167) |
| 8.08min 684.559m/z | 32.627 (39.242) | 26.320 (29.322) |
| 8.08min 449.313m/z | 51.540 (94.069) | 17.762 (4.167) |
| 8.09min 477.318m/z | 15.888 (0.516) | 24.365 (22.762) |
| 8.10min 448.312m/z | 469.798 (500.221) | 236.406 (242.954) |
| 8.10min 476.294m/z | 15.888 (0.516) | 22.904 (17.868) |
| 8.12min 297.244m/z | 135.953 (130.093) | 434.197 (656.057) |
| 8.15min 559.474m/z | 32.665 (39.661) | 51.606 (99.696) |
| 8.16min 682.542m/z | 28.347 (29.555) | 28.964 (41.803) |
| 8.16min 401.292m/z | 561.659 (562.837) | 418.974 (596.605) |
| 8.17min 702.575m/z | 64.668 (115.971) | 123.929 (98.035) |
| 8.18min 635.527m/z | 248.187 (257.140) | 68.963 (118.726) |
| 8.18min 597.518m/z | 15.888 (0.516) | 37.399 (49.408) |
| 8.18min 599.529m/z | 73.189 (106.669) | 67.935 (83.748) |
| 8.18min 822.601m/z | 20.529 (16.013) | 77.447 (61.969) |
| 8.19min 655.556m/z | 41.458 (88.850) | 133.738 (173.725) |
| 8.21min 568.338m/z | 35.467 (67.822) | 60.979 (104.687) |
| 8.21min 653.543m/z | 15.888 (0.516) | 29.776 (27.609) |
| 8.21min 637.540m/z | 112.383 (172.009) | 17.762 (4.167) |
| 8.21min 299.260m/z | 665.563 (1123.558) | 1471.674 (1143.788) |
| 8.21min 279.233m/z | 312.983 (376.734) | 239.634 (429.233) |
| 8.23min 281.249m/z | 132.382 (154.085) | 223.923 (713.560) |
| 8.24min 611.523m/z | 33.095 (44.742) | 17.762 (4.167) |
| 8.28min 255.233m/z | 68.480 (100.186) | 103.203 (295.392) |
| 8.29min 540.330m/z | 15.888 (0.516) | 27.045 (31.554) |
| 8.47min 313.238m/z | 44.652 (52.729) | 17.762 (4.167) |
| 8.61min 116.928m/z | 29.392 (46.831) | 17.762 (4.167) |
| 9.52min 235.926m/z | 15.888 (0.516) | 34.590 (41.272) |

Values are means and standard deviation (in bracket) of normalized intensities from each cultivation.

**Table S2** **Targeted organic acids per mycelial mass (mg/g) of *P. curvatum* AW02 from media with different carbon source**.

| Organic acids | Concentration (mg/g) | | | | | |
| --- | --- | --- | --- | --- | --- | --- |
|  | Media | | | | | |
|  | MSM-G | | | MSM-P | | |
| Pyruvic acid | 8 | ± | 2 | 2 | ± | 1 |
| Fumaric acid | 4 | ± | 1 | < LOD | | |
| Oxaloacetic acid | 115 | ± | 18 | 20 | ± | 6 |
| Malic acid | 159 | ± | 43 | 23 | ± | 7 |
| alpha-Ketoglutaric acid | 99 | ± | 0.0 | Trace | | |
| cis-Aconitic acid | 6 | ± | 1 | 2 | ± | 1 |
| Citric acid | 84 | ± | 9 | 24 | ± | 6 |
| Isocitric acid | Trace | | | Trace | | |
| Glyoxylic acid | Trace | | | Trace | | |
| Succinic acid | 21 | ± | 9 | 8 | ± | 2 |

Value are means ± standard deviation of three biological replicates. Each biological replicate has three technical replicate. <LOD; Below than limit of detection. Trace; Below than limit of quantitation. MSM-G; Mineral salt media-Glucose. MSM-P; Mineral salt media-Palm oil. The data had been fitted by removing tenths and hundreds of ppb and give in two largest decimal.

**Table S3** **Metabolic pathway analysis results.**

| Pathway name | Total^a^ | Hits^b^ | -log(p)^c^ | FDR | Impact^d^ |
| --- | --- | --- | --- | --- | --- |
| Alanine, aspartate and glutamate metabolism | 20 | 4 | 6.551 | 0.024 | 0.005 |
| Glycerophospholipid metabolism | 26 | 1 | 5.792 | 0.024 | 0.043 |
| Glyoxylate and dicarboxylate metabolism | 14 | 7 | 5.470 | 0.024 | 0.605 |
| Glycolysis or Gluconeogenesis | 24 | 2 | 4.929 | 0.025 | 0.099 |
| Starch and sucrose metabolism | 18 | 1 | 4.898 | 0.025 | 0.023 |
| Purine metabolism | 60 | 1 | 3.655 | 0.073 | 0.097 |
| Citrate cycle (TCA cycle) | 20 | 8 | 3.370 | 0.077 | 0.486 |
| Pyruvate metabolism | 23 | 3 | 3.322 | 0.077 | 0.307 |
| Arginine and proline metabolism | 37 | 1 | 2.854 | 0.109 | 0.000 |
| Glutathione metabolism | 23 | 1 | 0.000 | 1.000 | 0.429 |
| Glycine, serine and threonine metabolism | 26 | 1 | 0.000 | 1.000 | 0.000 |
| Cysteine and methionine metabolism | 33 | 1 | 0.000 | 1.000 | 0.000 |
| Valine, leucine and isoleucine biosynthesis | 24 | 1 | 0.000 | 1.000 | 0.000 |
| Tyrosine metabolism | 19 | 1 | 0.000 | 1.000 | 0.000 |
| Propanoate metabolism | 14 | 1 | 0.000 | 1.000 | 0.000 |
| Butanoate metabolism | 17 | 2 | 0.000 | 1.000 | 0.000 |
| Pantothenate and CoA biosynthesis | 16 | 1 | 0.000 | 1.000 | 0.000 |

^a^: the total number of metabolites in the pathway. ^b^: the actual matched number of metabolites from the uploaded data. ^c^: the logarithm of p value calculated from pathway enrichment analysis. ^d^: the pathway impact value calculated from pathway topology analysis.

**Table S4** **Linearity, regression equation, LOD and LOQ for each targeted organic acids**.

| Organic acids | Linearity |  | Regression equation | LOQ | LOD | Instruments |
| --- | --- | --- | --- | --- | --- | --- |
|  | Range | r^2^ |  |  |  |  |
| Pyruvic acid | 0.2-10 µg/mL | 0.99 | y = 0.171903x + 8.60854 | 0.2 µg/mL | 0.14 µg/mL | LC-MS/MS-TripleQ |
| Fumaric acid | 0.2-10 µg/mL | 0.99 | y = 7.6643x – 304.604 | 0.2 µg/mL | 0.15 µg/mL | LC-MS/MS-TripleQ |
| Oxaloacetic acid | 0.2-10 µg/mL | 0.99 | y = 0.290617x – 7.5684 | 0.2 µg/mL | 0.17 µg/mL | LC-MS/MS-TripleQ |
| Malic acid | 0.2-10 µg/mL | 0.99 | y = 3.7372x + 109.623 | 0.2 µg/mL | 0.15 µg/mL | LC-MS/MS-TripleQ |
| alpha-Ketoglutaric acid | 0.2-10 µg/mL | 0.99 | y = 2.58605x + 48.3575 | 0.2 µg/mL | 0.08 µg/mL | LC-MS/MS-TripleQ |
| cis-Aconitic acid | 0.2-10 µg/mL | 0.99 | y = 7.46825x + 84.238 | 0.2 µg/mL | 0.02 µg/mL | LC-MS/MS-TripleQ |
| Citric acid | 0.2-10 µg/mL | 0.99 | y = 11.8354x – 2254.45 | 0.2 µg/mL | 0.2 µg/mL | LC-MS/MS-TripleQ |
| Isocitric acid | 0.2-10 µg/mL | 0.99 | y = 0.100165x – 20.3101 | 0.2 µg/mL | 0.2 µg/mL | LC-MS/MS-TripleQ |
| Glyoxylic acid | 0.1-1 mg/mL | 0.96 | y = 996380x + 4E+07 | 100 mg/mL | 100 mg/mL | GCMS |
| Succinic acid | 0.2-10 µg/mL | 0.99 | y = 0.18206x – 16.8079 | 0.2 µg/mL | 0.2 µg/mL | LC-MS/MS-TripleQ |

LOQ: Limit of quantitation, LOD: Limit of detection.

**Table S5** **Recorded osmolality of distilled water, MSM-P medium and MSM-G medium, MSM-P culture and MSM-G culture.**

| **Sample** | **Osmolality, mOsm/kg** | | |
| --- | --- | --- | --- |
| Distilled water | 2 | ± | 1 |
| Media alone |  |  |  |
| MSM-P | 71.8 | ± | 7 |
| MSM-G | 91 | ± | 6 |
| Culture with *P. curvatum* AW02 |  |  |  |
| MSM-P | 67.2 | ± | 10 |
| MSM-G | 67 | ± | 2 |


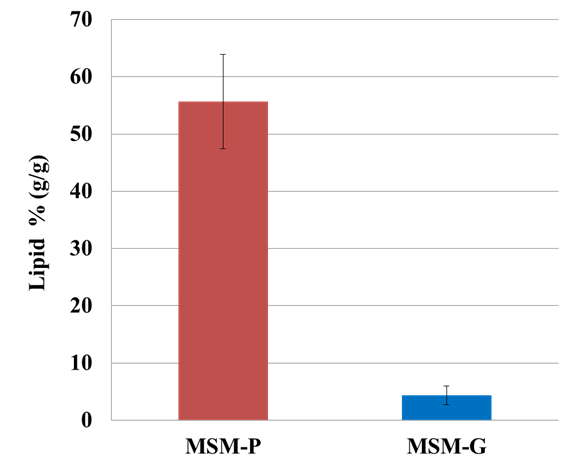


**Fig. S1 Lipid production against cells biomass (%) of *P. curvatum* cultured in MSM-P and MSM-G broth after 5 days**. Values are given as mean of 4 replicates. The data scattering were < 30%.


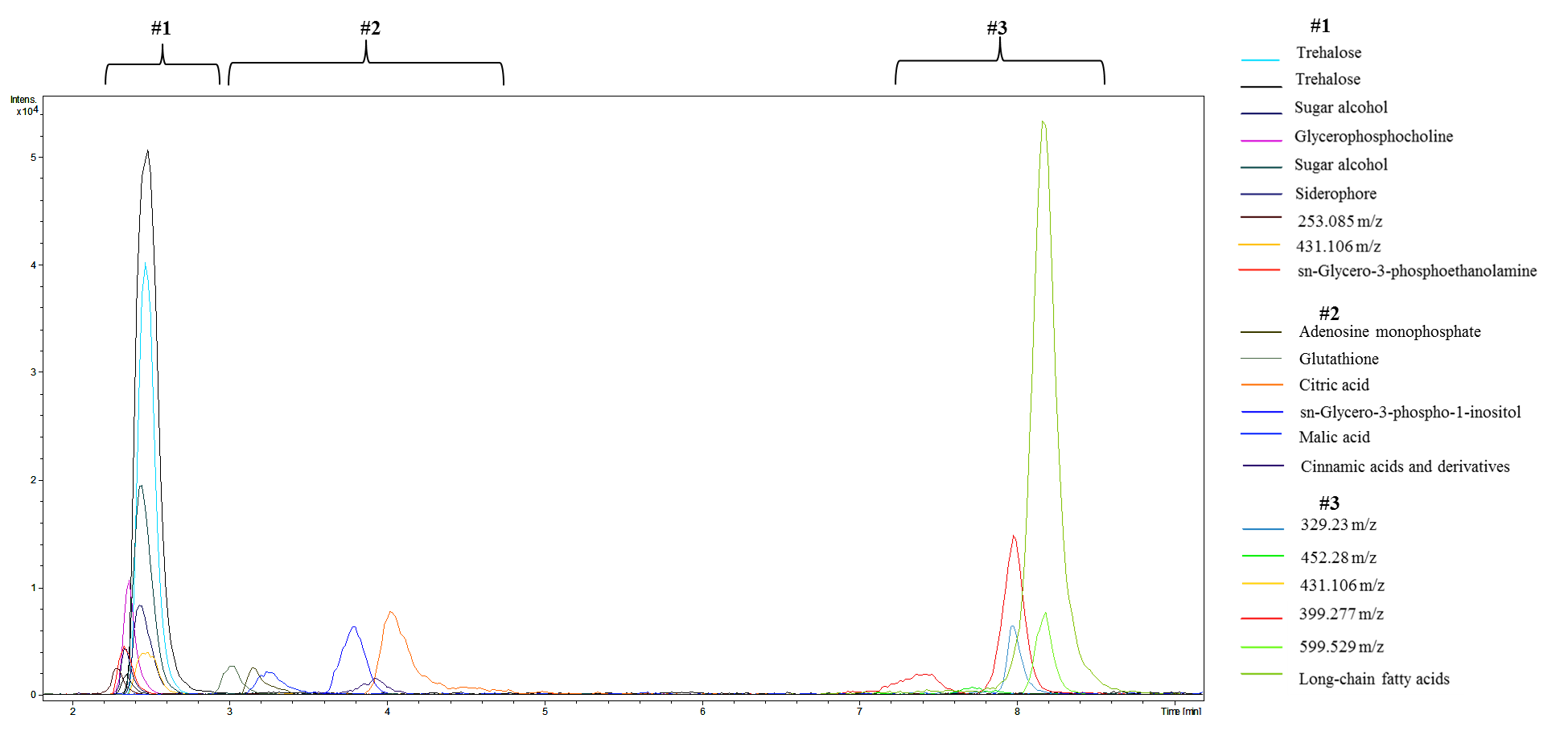


**Fig. S2** **Extracted Ion chromatograms (EIC’s) of each metabolites (level 1, 2 and 3) extracted from the biomass of *P. curvatum* AW02**. Unknown metabolites were named according their m/z values. Details of metabolites identification and their regulation between MSM-P and MSM-G are stated in **Table 1**. # number represent peak region according to retention time.


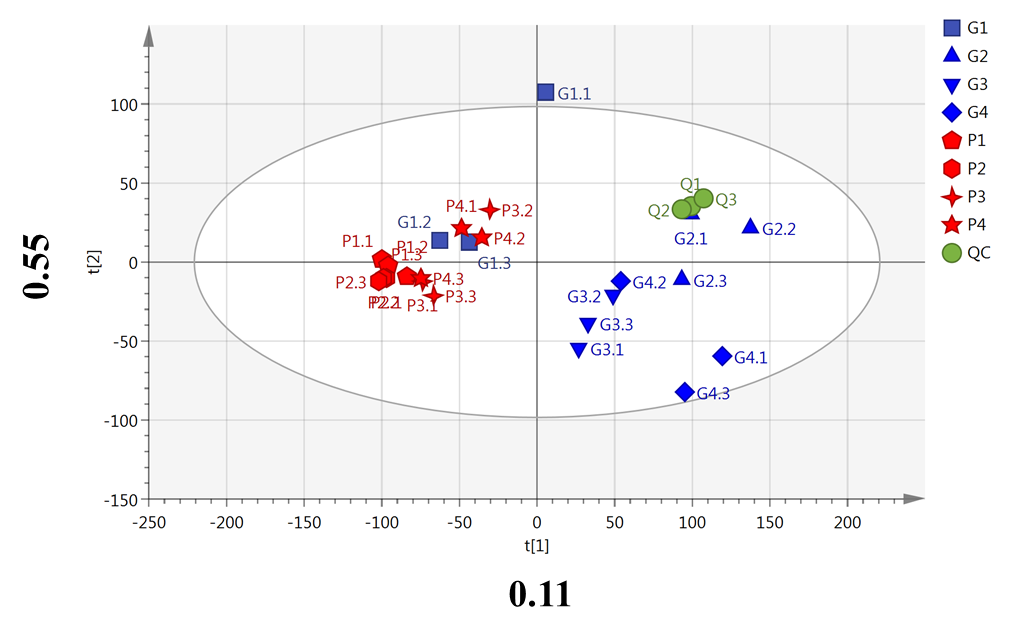


**Fig. S3** **PCA scores plot projection based on intracellular metabolite from MSM-P culture, MSM-G culture and pooled sample of MSM-P and MSM-G culture treated as a quality control (QC)**. Blue colour represents MSM-G, red colour represents MSM-P and green colour represents QC. Each shape represents a biological replicate, which consist of 3 technical replicates. The analysis showed less variation of QC which demonstrated the LC-MS-TOF system that been used was robust with good repeatability and stability.


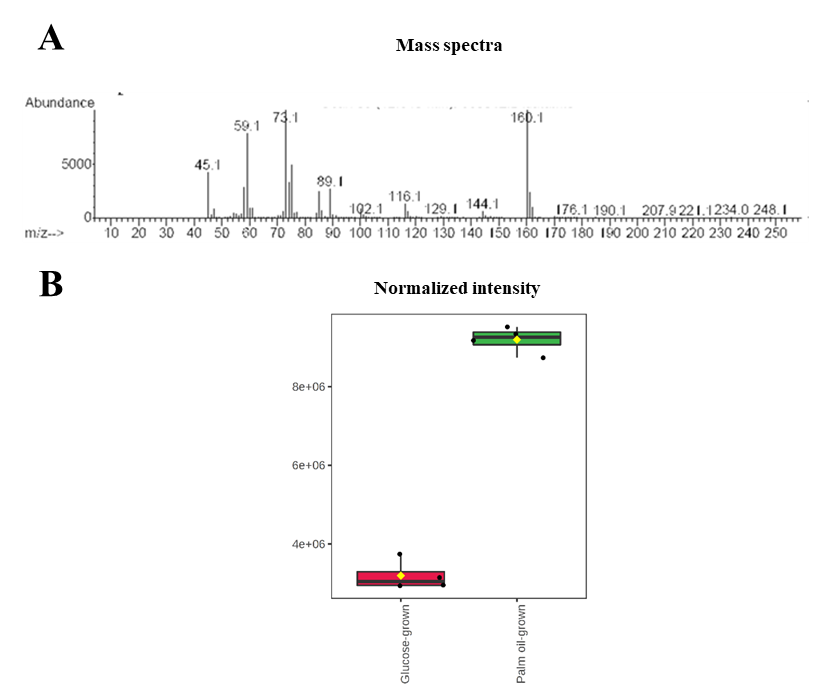


**Fig. S4** (**A) Mass spectra of glyoxylic acids standard undergo derivatization with trimethysily.** **(B) Comparative concentration of glyoxylic acid based on normalised intensity between palm oil-grown and glucose-grown.** The box and whisker plots summarized the normalized intensities of glyoxylic acid from each cultivation.

**
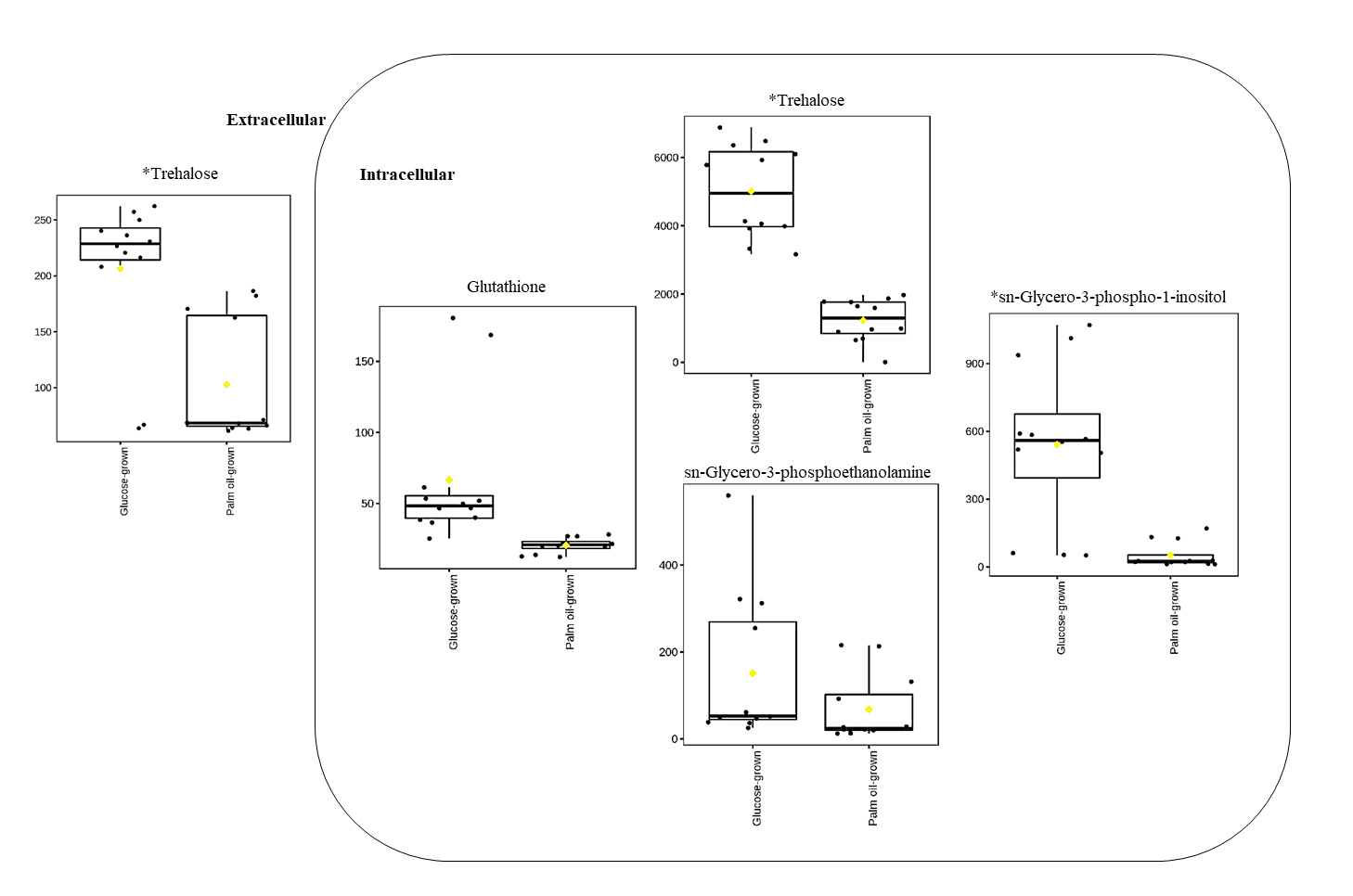
**

**Fig. S5** **Comparative concentration of different metabolites based on normalised intensity between palm oil-grown and glucose-grown**. The box and whisker plots summarized the normalized intensities of a metabolite from each cultivation.* Significant different in metabolite’s abundance between palm oil-grown and glucose-grown with VIP>0.95 and supported with p-value<0.01 and FDR cut-off 0.01.


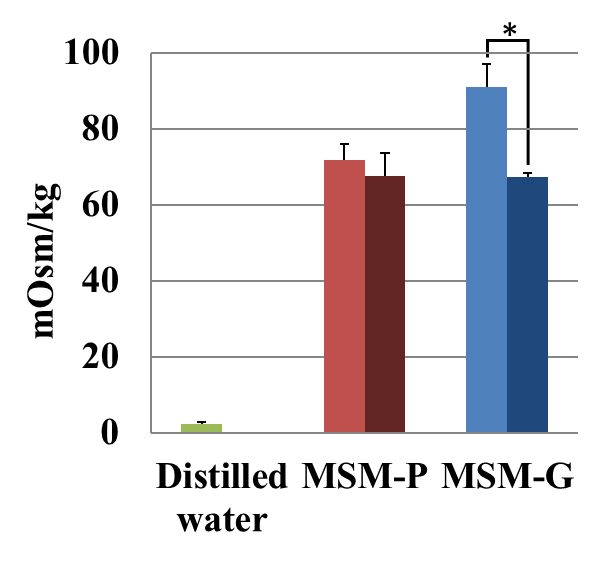


**Fig. S6** **The medium osmolality of distilled water (green), MSM-P (red) and MSM-G (blue)**. Light colour represents medium osmolality before culture started (media alone) while dark colour represents medium osmolality after 5-days culture period. Medium osmolality was measured in mOsm/kg using micro osmometer (Advanced Instrument, USA). * Significant different of osmolality between medium alone and culture at day-5 with p-value<0.05.


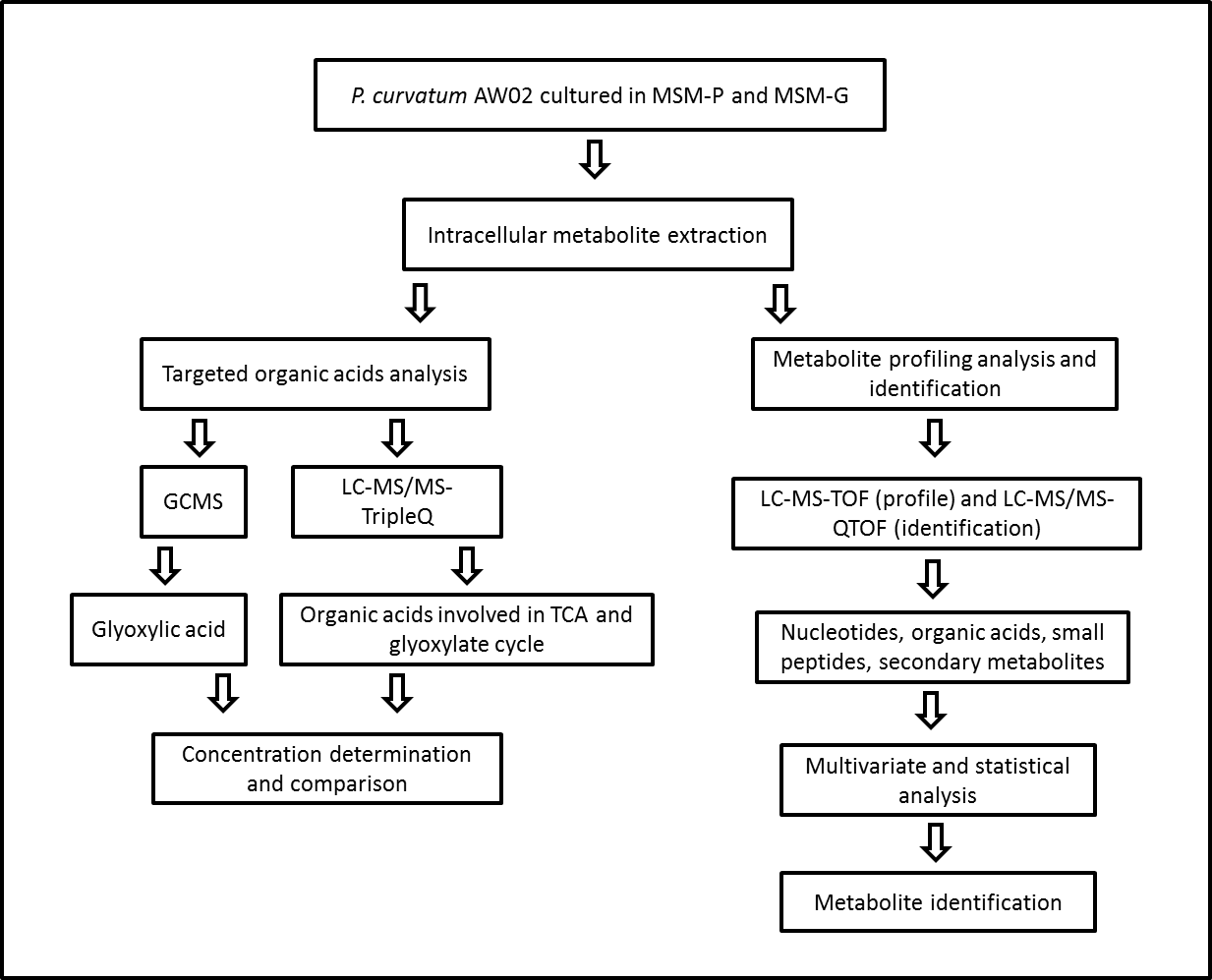


**Fig. S7** **Workflow for comparative analysis of *P. curvatum* AW02 cultured in MSM-P and MSM-G by using combination of GC-MS, LC-MS/MS-TripleQ and LC-MS/MS-QTOF.**


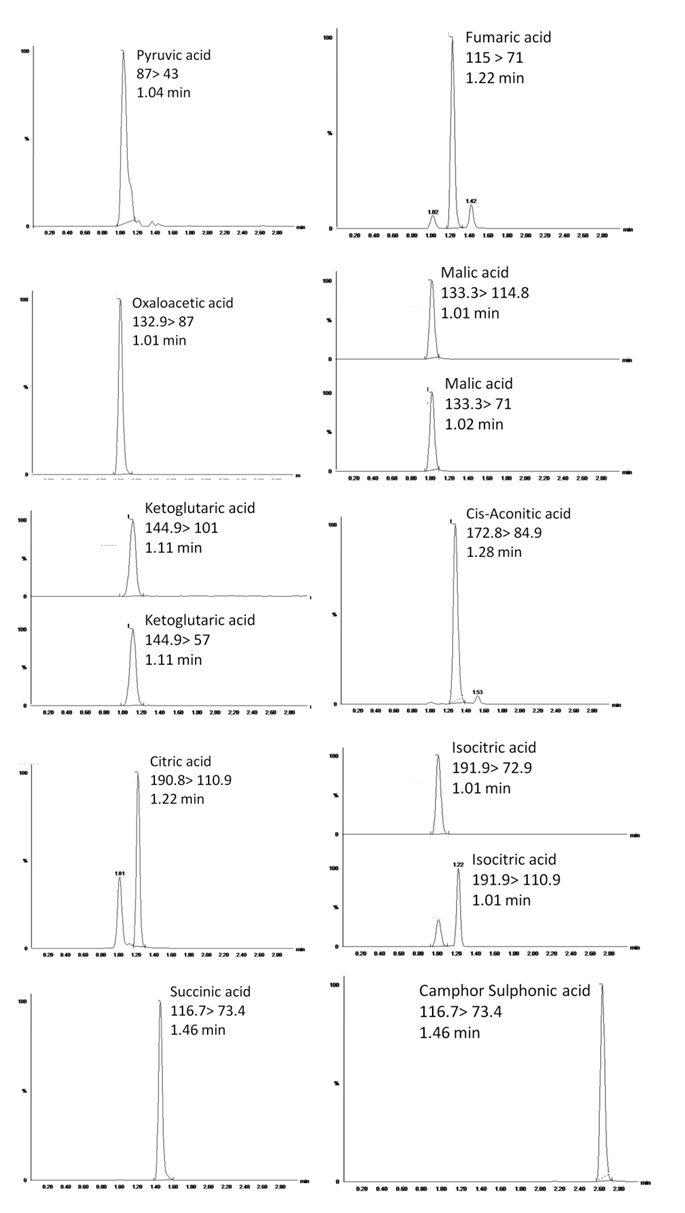


**Fig. S8** **Typical LCMS/MS chromatogram of organic acids standard.**
